# Selection in males purges the mutation load on female fitness

**DOI:** 10.1002/evl3.239

**Published:** 2021-06-26

**Authors:** Karl Grieshop, Paul L. Maurizio, Göran Arnqvist, David Berger

**Affiliations:** ^1^ Animal Ecology, Department of Ecology and Genetics Uppsala University Uppsala SE‐75236 Sweden; ^2^ Department of Ecology and Evolutionary Biology University of Toronto Toronto ON M5S 3B2 Canada; ^3^ Department of Molecular Biosciences The Wenner‐Gren Institute Stockholm University Stockholm SE‐10691 Sweden; ^4^ Section of Genetic Medicine, Department of Medicine University of Chicago Chicago Illinois 60637

**Keywords:** Diallel cross, fitness, good genes, heterosis, mutation load, sexual selection

## Abstract

Theory predicts that the ability of selection and recombination to purge mutation load is enhanced if selection against deleterious genetic variants operates more strongly in males than females. However, direct empirical support for this tenet is limited, in part because traditional quantitative genetic approaches allow dominance and intermediate‐frequency polymorphisms to obscure the effects of the many rare and partially recessive deleterious alleles that make up the main part of a population's mutation load. Here, we exposed the partially recessive genetic load of a population of *Callosobruchus maculatus* seed beetles via successive generations of inbreeding, and quantified its effects by measuring heterosis—the increase in fitness experienced when masking the effects of deleterious alleles by heterozygosity—in a fully factorial sex‐specific diallel cross among 16 inbred strains. Competitive lifetime reproductive success (i.e., fitness) was measured in male and female outcrossed F_1_s as well as inbred parental “selfs,” and we estimated the 4 × 4 male‐female inbred‐outbred genetic covariance matrix for fitness using Bayesian Markov chain Monte Carlo simulations of a custom‐made general linear mixed effects model. We found that heterosis estimated independently in males and females was highly genetically correlated among strains, and that heterosis was strongly negatively genetically correlated to outbred male, but not female, fitness. This suggests that genetic variation for fitness in males, but not in females, reflects the amount of (partially) recessive deleterious alleles segregating at mutation‐selection balance in this population. The population's mutation load therefore has greater potential to be purged via selection in males. These findings contribute to our understanding of the prevalence of sexual reproduction in nature and the maintenance of genetic variation in fitness‐related traits.

**Impact Statement**

Why do the large majority of eukaryotic species reproduce sexually if it means that females must spend half of their reproductive effort producing males, whereas males contribute few or no resources to offspring production themselves? In principle, a lineage of a mutant asexual female that simply clones herself into daughters would grow at twice the rate of her sexual competitors (all else equal). What prevents this from being the predominant mode of reproduction throughout eukaryotes? One hypothesis regards the role of males in facilitating the purging of deleterious mutations from the population's genome because very strong selection in males, unlike selection in females, can occur in many species without reductions in population offspring numbers. Due to the inherent difficulties of isolating this source of standing genetic variation for fitness, empirical evidence for this theory is mixed and limited to indirect evidence from manipulative experiments and experimental evolution studies. Here, we demonstrate that recessive deleterious alleles in a population of the seed beetle, *Callosobruchus maculatus*, are selected against in males but not females. Using a fully factorial diallel cross among 16 inbred strains, we measured the degree to which fitness in the outbred offspring of those crosses improved relative to their inbred parents. This measure is known as heterosis and offers an estimate of the relative amount of partially recessive deleterious alleles carried by a genetic strain. We then analyzed the relationship between the strains’ heterosis values and their additive genetic breeding values for fitness measured in males and females, revealing the extent to which segregating (partially recessive) deleterious alleles are selected against in males and females. We found that a strain's heterosis value was strongly genetically correlated with its additive genetic breeding value for male fitness, but not female fitness. This suggests that mutations with deleterious effects on population growth rate due to their effects on females can be selected against (i.e., purged) more efficiently via their male siblings. This process would offer a benefit to sexual reproduction that may partly compensate for its costs, and therefore yields insight to the prevalence of sex in nature.

Sexual reproduction is paradoxically prevalent when considering the cost of producing male offspring, which contribute little to offspring production themselves (Maynard Smith 1971, 1978; Lehtonen et al. 2012; Gibson et al. 2017). Counterintuitively, this same male feature may offer long‐term benefits to lineages producing sons, as deleterious alleles can be purged via strong selection in males without appreciable reductions to a lineage's offspring production (Manning 1984; Kodric‐Brown and Brown 1987; Agrawal 2001; Siller 2001; Whitlock 2002; Lorch et al. 2003; Whitlock and Agrawal 2009). For this process to advantage sexual lineages over mutant asexual competitors, and thereby account for the maintenance and prevalence of sex in eukaryotes, purifying selection against mutations with deleterious effects on female fecundity, and hence population offspring production, must be stronger in males than in females (Agrawal 2001; Siller 2001; Whitlock and Agrawal 2009). Selection is likely stronger in males than females in many systems (Wade and Arnold 1980; Whitlock and Agrawal 2009) owing to sexual selection operating more strongly in males (Wade 1979; Andersson 1994; Janicke et al. 2016), which is ultimately due to sex differences in gamete investment (i.e., anisogamy; Parker et al. 1972; Schärer et al. 2012).

Empirical support for male‐enhanced purging of the genetic load on females comes mostly from studies of induced, accumulated, or known mutations (e.g., Radwan 2004; Sharp and Agrawal 2008, 2013; Hollis et al. 2009; Grieshop et al. 2016) or experimental evolution (e.g., Firman and Simmons 2010, 2012; Lumley et al. 2015; Dugand et al. 2018, 2019b; Yun et al. 2018; Buzatto and Clark 2020; Kyogoku and Sota 2021; reviewed in Cally et al. 2019), but detecting this process in a snapshot of the standing genetic variation has proven difficult (Chenoweth et al. 2015). This difficulty may owe to interference between signals stemming from the “mutation load” and the “segregation load” (Crow 1958; Charlesworth and Charlesworth 2010; Whitlock and Davis 2011). The former is attributed to rare, often partially recessive, deleterious alleles in mutation‐selection balance (Haldane 1927; Lande 1975; Lynch et al. 1999; Zhang et al. 2004) and the latter is attributed to (net) heterozygote advantage, including genetic trade‐offs where heterozygotes are the most fit on average due to alleles having conditionally deleterious/beneficial effects on fitness (Rose 1982; Connallon and Clark 2012). The fact that mutation‐selection balance alone tends to be insufficient to account for all of the observed genetic variance in fitness and life history traits (Houle et al. 1994; Charlesworth and Hughes 2000; Barton and Keightley 2002; Mitchell‐Olds et al. 2007; Charlesworth 2015; Sharp and Agrawal 2018) suggests that the segregation load comprises a considerable fraction of a population's fitness variance. Thus, although sex‐specific estimates of variance in fitness would indicate the relative strength of selection in males versus females (Crow 1958; Charlesworth and Hughes 2000; Cox and Calsbeek 2009; Janicke et al. 2016; Singh and Punzalan 2018), simply comparing fitness variance between the sexes would confound the effects of rare unconditionally deleterious alleles with those that impose conditional fitness effects. This is particularly problematic for comparing the strength of purifying selection between the sexes in light of segregating sexually antagonistic alleles, whose fitness effects are conditional upon sex (Chippindale et al. 2001; Bonduriansky and Chenoweth 2009; van Doorn 2009; Connallon et al. 2010; Connallon and Clark 2012). This is because strongly selected male‐benefit/female‐detriment alleles that impose detriments to female offspring production will be overrepresented in the standing genetic variation relative to alleles with detrimental effects in both sexes that are maintained at mutation‐selection balance (Pischedda and Chippindale 2006; Long et al. 2012; Berger et al. 2016a). Indeed, this phenomenon is predicted to obfuscate experimental detection of the long‐term benefits of male‐enhanced purging of mutation load (Whitlock and Agrawal 2009, p. 576).

To address this issue, we experimentally uncovered sex‐specific fitness effects of the rare, partially recessive deleterious alleles that comprise the mutation load in a population of the seed beetle, *Callosobruchus maculatus*, by increasing genome‐wide homozygosity in 16 genetic strains and then analyzing heterosis for fitness (competitive lifetime reproductive success). Here, heterosis of a genotype is the increase in fitness in outbred progeny of crosses involving that genotype relative to its inbred/homozygous state (Charlesworth and Willis 2009). Although fitness variance (Charlesworth and Hughes 2000; Kelly and Willis 2001; Barton and Keightley 2002; Kelly 2003; Mitchell‐Olds et al. 2007; Charlesworth 2015; Sharp and Agrawal 2018) and inbreeding depression (Charlesworth and Charlesworth 1987, 1999; Charlesworth et al. 2007; Dugand et al. 2019a) can both be attributable to the mutation load and the segregation load, heterosis in crosses among inbred strains of a given population should be disproportionately attributable to the masking of rare, partially recessive deleterious alleles by heterozygosity (Charlesworth and Willis 2009). Our estimates of strain‐specific heterosis—the difference between a strain's outbred and inbred fitness—should therefore primarily reflect each strain's relative share of the population's mutation load on female offspring production. Although the segregation load may in theory also contribute to heterosis (Charlesworth and Willis 2009), it is unlikely to play a role in the present study population. First, the correlation between male and female heterosis is near unity (see *Results*), indicating that this population's most relevant source of segregation load, its sexually antagonistic genetic variation (Berger et al. 2014,2016a; Grieshop and Arnqvist 2018), contributes little or nothing to variation in heterosis among strains. Second, fitness in the inbred state exhibits a substantially reduced mean and increased variance relative to the outbred state (see *Results*), which is consistent with fitness being underlain by rare, partially recessive deleterious alleles (Robertson 1952; Houle et al. 1997; Kelly 1999; Charlesworth and Hughes 2000; Kelly and Tourtellot 2006).

We therefore reason that an approximation of the strength of purifying selection against mutation load alleles in each sex is given by quantifying the relationship between strains’ male and female additive genetic breeding values for relative fitness and their genetic values for heterosis (i.e., a measure that should reveal the relative amount of mutation load alleles carried by each strain). If selection in males acts to purge mutation load on female fecundity, there should be a negative genetic covariance between outbred male fitness and female heterosis (see *Methods*). In other words, strains with a greater relative share of the population's mutation load should have lower male fitness. Similarly, if selection in females acts to purge mutation load, there should be a negative genetic covariance between outbred female fitness and heterosis. Thus, we predicted a negative relationship between heterosis and outbred fitness among strains, and that this relationship would be stronger in males than females if selection in males enhances the purging of mutation load.

## Methods

### STUDY POPULATION AND INBRED STRAINS

*Callosobruchus maculatus* (Coleoptera: Bruchidae) is a pest of leguminous crops that has colonized most of the tropical and subtropical regions of the world (Southgate 1979). Laboratory conditions and fitness assays closely resemble the grain storage facilities and crop fields to which they are adapted. Females lay eggs on the surface of dry beans and hatched larvae bore into the beans, where they complete their life cycle, emerging from the beans as reproductively mature adults (Southgate 1979). This species is facultatively aphagous (requiring neither food nor water to reproduce successfully), exhibits a generation time of ∼3 weeks (Southgate 1979), and exhibits a polyandrous mating system (Miyatake and Matsumura 2004; Katvala et al. 2008).

The origin of this population's isofemale lines and inbred lines has been described thoroughly by Berger et al. (2014), Grieshop et al. (2017), and Grieshop and Arnqvist (2018). Briefly, 41 isofemale lines were constructed from a wild population of *C. maculatus* that was isolated from *Vigna unguiculata* seed pods collected at a small‐scale agricultural field close to Lomé, Togo (06°10'N 01°13'E) during October and November 2010 (Berger et al. 2014; see Fig. S1A). Each isofemale line stemmed from a single virgin male/female pair whose offspring were expanded into small subpopulations (Fig. S1A). These 41 isofemale lines have an inbreeding coefficient of 0.25 (Falconer and Mackay 1996) and were cultured for 12 generations prior to the fitness assays of Berger et al. (2014). From January 2013 to January 2014, 20 replicate lineages of each isofemale line (totaling >800 lineages) were subjected to single‐pair full‐sibling inbreeding (i.e., “close” inbreeding; Falconer and Mackay 1996) for 10 consecutive generations or until extinction (Grieshop et al. 2017; Fig. S1A). For the 16 inbred strains chosen for the present diallel cross, this was followed by one generation of expansion into small populations, comprising a total of 12 generations of full‐sibling mating (1 full‐sibling isofemale line expansion + 10 generations of close inbreeding + 1 full‐sibling inbred line expansion), which corresponds to an inbreeding coefficient of 0.926 (Falconer and Mackay 1996). During the inbreeding regime, inbred lineages stemming from isofemale lines that were enriched for male‐benefit/female‐detriment sexually antagonistic genetic variation tended to go extinct prior to completing the full inbreeding program (Grieshop et al. 2017), making it impossible to retrieve a representative inbred line from four of the most male‐benefit/female‐detriment isofemale lines. The present 16 inbred strains were chosen with the aim of countering that biased representation (Fig. S2).

### SEX‐SPECIFIC FITNESS ASSAY

The present study used data from a fully factorial diallel cross (Lynch and Walsh 1998) among the 16 inbred strains, where sex‐specific competitive lifetime reproductive success (hereafter: fitness) was measured in F_1_ males and females separately. The partitioning of genetic variance in fitness is reported in detail by Grieshop and Arnqvist (2018), and the aspects that bear relevance to the present study are given below and in the *Discussion*. The experiment was conducted in two replicate “blocks” for a total of 3278 individual fitness estimates performed in 237 of 240 possible outbred crosses and all 16 parental selfs (Fig. S1B). Male and female fitness, as well as inbred and outbred fitness, was measured independently (Fig. S1B,C), and all were approximately normally distributed. There are 1616 outbred male ($o_{M}$), 1450 outbred female ($o_{F}$), 115 inbred male ($i_{M}$), and 97 inbred female ($i_{F}$) individual fitness estimates. Each observation of the fitness assay consisted of a 90‐mm ø petri dish containing about 100 *V. unguiculata* seeds, a focal individual from a given outbred cross or inbred self, a sterilized same‐sex competitor from the outbred base population, and two opposite‐sex individuals from the base population (Fig. S1C). Same‐sex competitors were sterilized with a 100 Gy dose of ionizing radiation from a Cs^137^ source (Fig. S1C), which does not notably reduce life span or reproductive competitiveness in either sex, but does cause zygote lethality, accrediting all emerging offspring to focal individuals (Grieshop et al. 2016). Thus, counts of F_2_ offspring emerging in the petri dishes are integrative measures of focal individuals’ fitness (Fig. S1C), the differences among them being attributable to focal F_1_ individuals’ pre‐ and postcopulatory reproductive success and their offspring's larval viability. Our fitness assays therefore enable, but are not limited to, the following mechanisms of selection in adults: mate searching and oviposition site selection, mating success, mating resistance, sexual conflict over remating rate, sperm competition, cryptic female choice, fertility, fecundity, life span, and offspring egg‐adult survival (discussed further by Grieshop et al. 2016; Grieshop and Arnqvist 2018). Despite lacking many of the elements of natural selection that might apply to these beetles in nature (as would any laboratory fitness assay), this method has been effective in revealing sex‐specific genetic variance in fitness (Berger et al. 2014, 2016a,b; Grieshop et al. 2016; Martinossi‐Allibert et al. 2019b), perhaps owing to its relatively greater resemblance to the beetles’ natural ecology compared with other model systems and/or the inherent three‐dimensional physical complexity provided by the beans, which may play an important role in achieving balance between sexually concordant and sexually antagonistic mating interactions in the laboratory (Singh et al. 2017; Yun et al. 2017).

### MODELING THE GENETIC COVARIANCE MATRIX

We modeled the male‐female‐inbred‐outbred additive genetic (co)variance matrix for fitness, H:

(1) $$H\;=\left[\begin{array}{cccc} i_{F} & i_{F},o_{F} & i_{F},i_{M} & i_{F},o_{M}\\ o_{F},i_{F} & o_{F} & o_{F},i_{M} & o_{F},o_{M}\\ i_{M},i_{F} & i_{M},o_{F} & i_{M} & i_{M},o_{M}\\ o_{M},i_{F} & o_{M},o_{F} & o_{M},i_{M} & o_{M} \end{array}\right],$$

where genetic variances were estimated for parameters listed along the diagonal, and genetic covariances were estimated for pairs of parameters listed in the off‐diagonal elements. Although this resembles a cross‐sex cross‐trait G matrix that would typically be modeled with the aim of assessing the sex‐specific genetic architecture of multiple traits and whether it constrains or enables (sex‐specific) adaptation (Lande 1980; Gosden and Chenoweth 2014; Ingleby et al. 2014; McGlothlin et al. 2019; Sztepanacz and Houle 2019; Cheng and Houle 2020; Kollar et al. 2021), our H matrix is distinct in two important ways: (1) it is a cross‐sex cross‐*state* (rather than cross‐trait) (co)variance matrix, which models inbred/homozygous effects versus outbred/heterozygous effects for the same “trait,” and (2) that “trait” is fitness.

The H matrix was modeled in a general linear mixed‐effects model (GLMM) using Bayesian Markov chain Monte Carlo (MCMC) simulations in the “MCMCglmm” package (version 2.25; Hadfield 2010) for R (version 3.6.0; R Core Team 2019). To attain proper estimates of additive genetic variance, two additional random effects (and corresponding variance components) were included to estimate symmetrical epistasis (v) and sex‐specific symmetrical epistasis (v×S) (i.e., [sex‐specific] strain‐strain interaction variance among outcrossed families only), as these effects are known to be present in these data (Grieshop and Arnqvist 2018). Fixed factors in this model were sex (S, male or female), inbred (I, inbred self or outbred cross), block (B, first or second replicate of the full diallel cross), and the interactions S×I and B×I, making the full GLMM:

(2) y=μ+S+B+I+S×I+B×I+H+v+v×S+ε,

where y is relative fitness, μ is the intercept, and ε is the residual/unexplained error, normally distributed as $\varepsilon ∼N(0,\;\sigma^{2})$ with variance $\sigma^{2}$. We enabled (co)variance estimation to differ among elements of the H matrix, and used minimally informative parameter‐expanded priors (Hadfield 2012). The model was run for 2,000,000 iterations after a burn‐in of 200,000, with a thinning interval of 2000, which provided 1000 uncorrelated posterior estimates of each sex‐/strain‐specific effect to be stored and used for resampling the relationships described below. We used the Gelman‐Rubin criterion to ensure model convergence (Gelman and Rubin 1992; Fig. S3). Posteriors were unimodally distributed and their trend was stable over the duration of the simulations after the burn‐in period. The model that was fit for the purpose of estimating the H matrix and resampling the stored posteriors (see below) was fit to relative fitness, i.e., fitness standardized by the sex‐specific outbred mean, whereas the model that was fit for the purpose of plotting results was fit to untransformed/raw data.

We estimated heterosis (e.g., in females) as the difference between a strain's outbred, $o_{F}$, and inbred, $i_{F}$, fitness. As explained in the *Introduction* and in more detail below, the genetic variance in female heterosis, $V(o_{F}-i_{F})$, and its genetic covariance with male outbred fitness, $COV(o_{M},o_{F}-i_{F})$, are of central interest to our study, but are not explicitly modeled in our H matrix. Relationships involving heterosis were therefore assessed by resampling the sex‐/state‐specific breeding values from the 1000 stored posteriors. This approach incorporates the uncertainty around those breeding values into the estimated relationships and their credibility intervals (CIs), hence enabling the quantification of statistical significance in a way that accounts for breeding values being otherwise anticonservative when used to assess relationships that are not accounted for by the model (Postma 2006; Hadfield et al. 2010).

### ESTIMATING GENETIC RELATIONSHIPS BETWEEN OUTBRED FITNESS AND HETEROSIS

Assuming that heterosis is predominantly due to rare (partially recessive) deleterious alleles (see *Introduction* section and *Discussion*), we develop two complementary measures of how such mutation load alleles affect outbred fitness. Our first measure approximates the strength of selection on partially recessive deleterious alleles ($\beta_{a}^{\prime }$). In males, for example, $\beta_{a_{M}}^{\prime }$ is given by the genetic covariance between outbred relative fitness and female heterosis, divided by the genetic standard deviation in heterosis:$\;\;\beta_{a_{M}}^{\prime }=\;COV\left(o_{M},o_{F}-i_{F}\right)/SD\left(o_{F}-i_{F}\right)$. We note that our measure of selection is an additive genetic version (hence the subscripted “a”) of a univariate standardized phenotypic selection gradient ($\beta_{z}^{\prime }=\;\mathrm{Cov}\left[\omega ,z\right]/\sigma_{z}$; Lande and Arnold 1983, p. 1219), or “selection intensity” (Crow 1958; Falconer and MacKay 1996), used to provide comparable estimates of the strength of phenotypic selection across different traits or populations (Matsumura et al. 2012; Walsh and Lynch 2018). Our usage of this formulation differs from its original application in that our “phenotype,” heterosis, is not a feature of individuals but rather of genetic strains, and hence, our estimate of selection intensity is based on genetic (co)variances as advocated by Rausher (1992). This estimate—being standardized by $SD(o_{F}-i_{F})$—has an advantage to other measures of selection as it is independent of the arbitrary magnitude of heterosis in our population, which is a direct consequence of the number of generations of inbreeding that we applied in our experiment. Thus, $\beta_{a}^{\prime }$ gives the genetic change in outbred relative fitness associated with one genetic standard deviation change in heterosis, which reflects the amount of mutation load alleles in the present population. Further, the comparison of this standardized measure of selection in males ($\beta_{a_{M}}^{\prime }$) versus females ($\beta_{a_{F}}^{\prime }$, defined below) estimates the sex difference in selection against these mutation load alleles. Retrieving unbiased estimates of $\beta_{a_{F}}^{\prime }$ is problematic, however, due to measurement error in female outbred fitness also featuring in the heterosis term of both the numerator and denominator, which could drive spurious correlations and false positive discoveries (Postma 2011; Berger and Postma 2014). To address this, we explored whether male estimates of heterosis ($o_{M}-i_{M}$) may be so highly genetically correlated to the female estimates ($o_{F}-i_{F}$) that they effectively convey the same information, enabling $\beta_{a_{F}}^{\prime }$ to be estimated using male heterosis. As male and female heterosis were, indeed, highly correlated (see *Results*), we ultimately estimated selection in males and females as

(3a) $$\beta_{a_{M}}^{\prime }=\;COV\left(o_{M},o_{F}-i_{F}\right)/SD\left(o_{F}-i_{F}\right)$$

and

(3b) $$\beta_{a_{F}}^{\prime }=\;COV\left(o_{F},o_{M}-i_{M}\right)/SD\left(o_{M}-i_{M}\right),$$

respectively.

The genetic correlation between male and female heterosis, $r_{oM-iM,oF-iF}$ (used to assess the validity of 3a and 3b), is also informative regarding the degree to which the deleterious effects of the genetic variation underlying heterosis in our population are conditional upon sex or not, where $r_{oM-iM,oF-iF}=\;1$ would indicate that heterosis is attributable to alleles whose effects are completely unconditional on sex and correlations below unity would indicate some sex‐specificity to heterosis. Thus, the following genetic correlation was resampled 1000 times from the stored posteriors:

(4) $$r_{o_{M}-i_{M},o_{F}-i_{F}}=\frac{COV\left(o_{M}-i_{M},o_{F}-i_{F}\right)}{\sqrt{V\left(o_{M}-i_{M}\right)\;\cdot \;V\left(o_{F}-i_{F}\right)}}\;.$$

However, even though $o_{M}-i_{M}$ and $o_{F}-i_{F}$ were highly genetically correlated (see *Results*), $\beta_{a_{M}}^{\prime }\;$and $\beta_{a_{F}}^{\prime }$ are not directly comparable. Thus, the assessment of whether $\beta_{a_{M}}^{\prime }\;$and $\beta_{a_{F}}^{\prime }$ were significantly different, as well as the magnitude of their fold difference, was assessed using sex‐averaged heterosis, that is, $\beta_{a_{M}}^{{}^{\prime \prime }}=\;COV\left(o_{M},o-i\right)/SD\left(o-i\right)$ and $\beta_{a_{F}}^{{}^{\prime \prime }}=\;COV\left(o_{F},o-i\right)/SD\left(o-i\right)$, where $o-i\;=\left(\frac{o_{M}+o_{F}}{2}\right)-\left(\frac{i_{M}+i_{F}}{2}\right)$. Despite the potential bias owing to shared measurement error (described above), $\beta_{a_{M}}^{{}^{\prime \prime }}$ and $\beta_{a_{F}}^{{}^{\prime \prime }}$ provided a qualitatively identical result to $\beta_{a_{M}}^{\prime }$ and $\beta_{a_{F}}^{\prime }$, respectively (see Table S1), but enable a like‐to‐like comparison that is our least caveated estimate of the sex‐difference in the efficacy of purifying selection against partially recessive deleterious alleles.

Our second estimate of how these rare partially recessive deleterious alleles affect fitness is given by the genetic correlation between outbred fitness and heterosis. The issue with shared measurement error in female outbred fitness and heterosis also applies to these correlations, and we thus took the same approach as described above when estimating the male and female correlations:

(5a) $$r_{o_{M},o_{F}-i_{F}}=\frac{COV\left(o_{M},o_{F}-i_{F}\right)}{\sqrt{V\left(o_{M}\right)\;\cdot \;V\left(o_{F}-i_{F}\right)}}\;$$

and

(5b) $$r_{o_{F},o_{M}-i_{M}}=\frac{COV\left(o_{F},o_{M}-i_{M}\right)}{\sqrt{V\left(o_{F}\right)\;\cdot \;V\left(o_{M}-i_{M}\right)}}\;,$$

respectively. These correlations are merely different standardizations of the same numerator as the $\beta_{a}^{\prime }$ estimates (above); hence, their predictive frameworks (and reasoning therein) are similar to that given above, but with some important differences. Most importantly, they no longer reflect the strength of selection, per se, as they are standardized by fitness variance. Thus, these correlations represent the extent to which outbred fitness variance reflects mutation load alleles in each sex, where a correlation of −1 suggests that all fitness variance is solely due to rare and unconditionally deleterious alleles. These correlations are thus of particular interest for sexual selection theories of mate choice (Zahavi 1975; Hamilton and Zuk 1982; Grafen 1990; Andersson 1994) and “genic capture” in sexually selected traits (Rowe and Houle 1996; Tomkins et al. 2004), the latter based on the specific assumption that variance in sexually selected traits is maintained by polygenic deleterious mutation (see *Discussion*). Again, sex‐averaged heterosis (see above) was used to assess whether male and female genetic correlations were significantly different from one another. Our main interpretations are therefore based on the estimated selection intensities (equations 3a and 3b) and genetic correlations (equations 5a and 5b), where sex differences in those were assessed using sex‐averaged heterosis. All frequently used symbols are listed in Table A1.

Note that the selection intensities and correlations that were based on sex‐averaged heterosis not only feature shared measurement error between outbred fitness and heterosis, but potentially also shared MCMC sampling error upon resampling these estimates from the posteriors. To avoid this issue, each of the 1000 resampled estimates of sex‐averaged selection intensities and correlations drew their vector of sex‐specific outbred fitness estimates ($o_{M}$ and $o_{F}$) from different iterations than those used for sex‐averaged heterosis (“o−i”). The point estimates of selection intensities and genetic correlations are the posterior mode of the Bayesian posterior distribution based on 1000 resampled estimates, and these distributions were unimodal in all cases. The (95%) CIs around those point estimates are given by the highest posterior density (HPD) intervals. Two‐tailed *P*‐values for these correlations and covariances were calculated as the proportion of times that those 1000 estimates fell on the opposite side of zero relative to the posterior mode (or overlapped the point estimate of the other sex in the case of assessing sex differences), multiplied by two. The plotted breeding values, heterosis estimates, and 95% confidence ellipses are for visual purposes only, and are based on the HPD means of the model fit of untransformed/raw fitness; they do not depict the uncertainty in those breeding values that was incorporated into the resampled estimates of CIs and *P*‐values. Because these HPD means are zero‐centered in the “MCMCglmm” output (even for models fit to untransformed data), they were rescaled to the more intuitive original scale before plotting, and the minimum heterosis value was set to zero.

The potential for nongenetic parental effects and sex‐chromosome inheritance to explain our findings was thoroughly addressed. In short, we statistically removed these effects from our data and reran our analyses to confirm that our findings stand in the absence of those effects (Appendix B). See *Data archiving* regarding the R code for reproducing all analyses, procedures, tables, and figures.

## Results

The genetic variance in fitness for inbred males, $V(i_{M})$, was 1.85× that of inbred females, $V(i_{F})$, and the genetic variance for outbred males, $V(o_{M})$, was 1.24× that of outbred females, $V(o_{F})$ (Table 1). Males also exhibited 3.37× and 5.58× the residual variance in fitness relative to females when inbred and outbred, respectively (Table 1). Genetic variance for inbred fitness was 10.92× and 7.35× that of outbred fitness in males and females, respectively (Table 1).

**Table 1:** Estimated H matrix for relative fitness, displaying genetic variances (with credibility intervals [CIs] and residual variances) on the diagonal, their covariances in the lower triangle, and their Pearson's correlation coefficients (*r*) in the upper triangle. Covariances and correlations with 95% CIs excluding zero are bolded

	*i_F_*	*o_F_*	*i_M_*	*o_M_*
*i_F_*	0.0108	0.43	**0.85**	0.46
	(0.004, 0.021)			
	(0.0256)			
*o_F_*	0.001	0.0015	0.20	0.03
		(<0.001, 0.003)		
		(0.0150)		
*i_M_*	**0.0106**	0.0002	0.0199	**0.67**
			(0.006, 0038)	
			(0.0863)	
*o_M_*	0.0016	−2.5 × 10^–5^	**0.0030**	0.0018
				(<0.001, 0.004)
				(0.0837)

**Figure 1:**
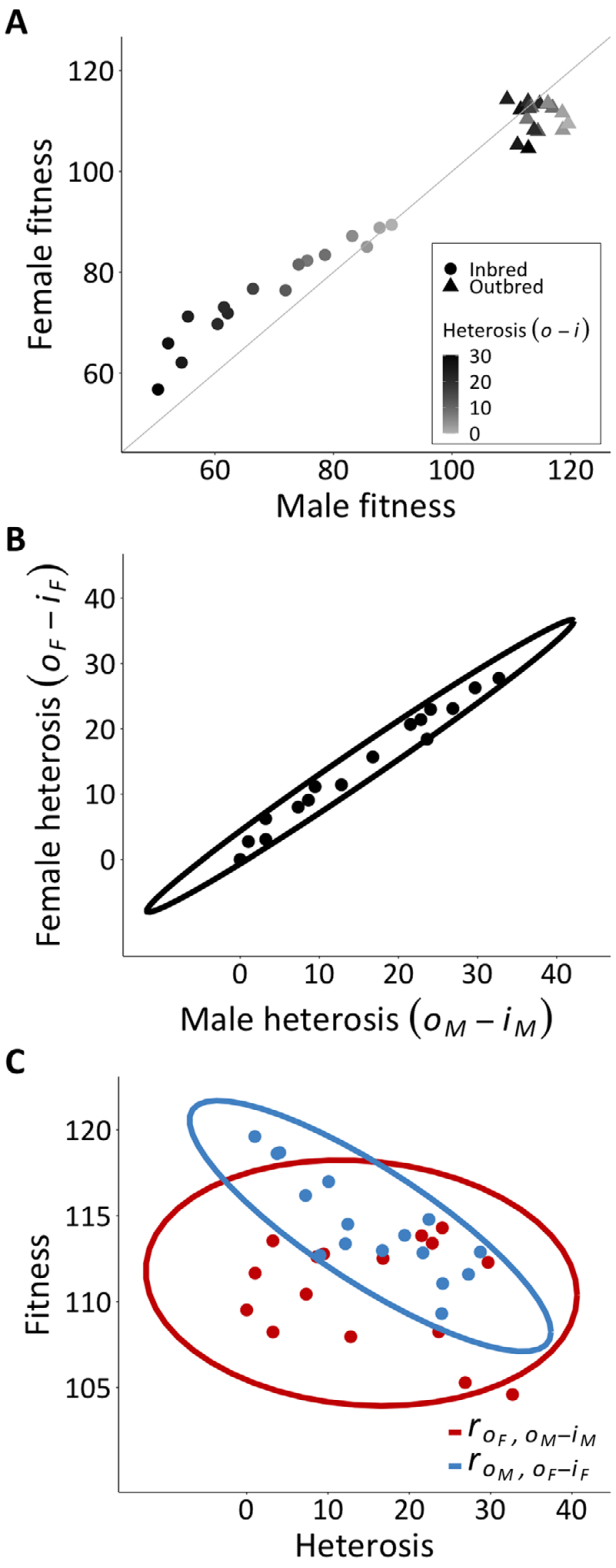
Breeding values from the MCMC model of untransformed (raw) fitness (for plotting purposes only). (A) A summary of the data and main result (*y* = *x* line for reference), showing each strain's male (*x*‐axis) and female (*y*‐axis) fitness in the inbred (circles: $r_{i_{M},\;\;i_{F}}$ = 0.85 [95% CI, 0.38, 0.97]) and outbred (triangles: $r_{o_{M},\;\;o_{F}}$ = 0.03, [−0.56, 0.54]) state, shaded by sex‐averaged heterosis (i.e., {[*o_M_* – *i_M_*] + [*o_F_* – *i_F_*]}/2). Variation in heterosis is clearly distributed along the population's outbred male, but not female, breeding values. (B) Depiction of the genetic correlation for heterosis in male and female fitness across strains, showing that these sex‐specific measures are conveying essentially the same information ($r_{o_{M}-i_{M},\;\;o_{F}-i_{F}}$ = 0.86 [95% CI, 0.66, 0.95], *P* < 0.001). (C) Depiction of main finding: the statistically significant resampled genetic correlation between outbred male fitness and female heterosis (blue: $r_{o_{M},\;\;o_{F}-i_{F}}$ = −0.59 [95% CI, −0.81, −0.11], *P* = 0.008) would enable the mutation load on population mean fitness to be purged via selection in males. In contrast, the outbred female breeding values do not reflect this mutation load (red: $r_{o_{F},\;\;o_{M}-i_{M}}$ = −0.14 [95% CI, −0.40, 0.28], *P* = 0.672). Ellipses are 95% confidence ellipses fit to the breeding values, and therefore do not depict the uncertainty that was included in the resampled estimates of statistical significance (see *Methods*). $\beta_{a_{M}}^{\prime }$ and $\beta_{a_{F}}^{\prime }$ depicted in Fig. S6.

There was a large global improvement in mean fitness of outcrossed observations relative to the inbred/homozygous parental selfs (i.e., the fixed effect of *I*, mean reduction in relative fitness of inbreds versus outbreds = 0.294 [95% CI, 0.19, 0.41], PMCMC < 0.001; Fig. 1A). Although outbred fitness in males and females was genetically uncorrelated ($r_{o_{M},\;\;o_{F}}$ = 0.03 [95% CI, −0.56, 0.54]; Figs. 1A and S4), inbred fitness was highly genetically correlated ($r_{i_{M},\;\;i_{F}}$ = 0.85 [95% CI, 0.38, 0.97]; Figs. 1A and S5), suggesting that these large heterosis effects were unconditional with respect to sex (estimated directly below). These correlations are explicitly estimated in the H matrix (Table 1).

Heterosis was highly genetically correlated between males and females (resampled $r_{o_{M}-i_{M},\;\;o_{F}-i_{F}}$ = 0.86 [95% CI, 0.66, 0.95], *P* < 0.001; Fig. 1B) with even narrower CIs than the inbred intersexual genetic correlation, $r_{i_{M},\;\;i_{F}}$ (see above), showing alignment between the sexes in the deleterious effects revealed by heterosis among strains. The point estimate of the strength of selection in males against these partially recessive deleterious effects on female fitness showed that one genetic standard deviation change in heterosis was associated with 1.25% reduction in outbred male fitness (resampled $\beta_{a_{M}}^{\prime }$ = −0.0125 [95% CI, −0.031, −0.003], *P* = 0.008; Fig. S6). By contrast, the corresponding estimate of selection in females was weak and undetectable (resampled $\beta_{a_{F}}^{\prime }$ = −0.0014 [95% CI, −0.013, 0.009], *P* = 0.672; Fig. S6). Outbred male fitness was significantly and strongly negatively genetically correlated to female heterosis (resampled $r_{o_{M},\;\;o_{F}-i_{F}}$ = −0.59 [95% CI, −0.81, −0.11], *P* = 0.008; Fig. 1C), suggesting that a sizeable proportion of fitness variance in males is due to rare partially recessive deleterious alleles. Female fitness, by contrast, was not significantly genetically correlated to male heterosis (resampled $r_{o_{F},\;\;o_{M}-i_{M}}$ = −0.14 [95% CI, −0.40, 0.28], *P* = 0.672; Fig. 1C). Estimates of selection and genetic correlations that were based on sex‐averaged heterosis yielded qualitatively identical results (Table S1). Using those directly comparable estimates based on sex‐averaged heterosis revealed that selection against partially recessive deleterious alleles was 3.7× stronger in males than females, yet estimates of selection (proportion of 1000 estimates of $\beta_{a_{F}}^{{}^{\prime \prime }}$ > $\beta_{a_{M}}^{{}^{\prime \prime }}$ times two: *P* = 0.104) and genetic correlations (*P* = 0.12) were not significantly different between the sexes using two‐sided hypothesis testing.

## Discussion

Our findings suggest that the mutation load of our population is more effectively purged via selection in males than in females (Fig. 1C). With heterosis being so highly sexually concordant (Fig. 1B), we were able to circumvent the potential bias caused by shared measurement error between fitness and heterosis by assessing the relationship between the outbred breeding values in one sex and heterosis in the other (see *Methods*, equations 3a,b and 5a,b). Moreover, relationships between male fitness and female heterosis ($\beta_{a_{M}}^{\prime }\;$ and $r_{o_{M},\;\;o_{F}-i_{F}}$; see equations 3a and 5a) are much more central to the question of whether selection via males can purge a population's mutation load, because population productivity in most taxa is limited by female offspring production. That the female equivalents of this assessment ($\beta_{a_{F}}^{\prime }$ and $r_{o_{F},\;\;o_{M}-i_{M}}$; see equations 3b and 5b) were found to be indistinguishable from zero suggests that genetic variation in fitness among females of this population does not evidently reflect the partially recessive deleterious mutations that they carry in their genomes. These results remained essentially unchanged when analyses were performed on estimates of heterosis averaged across the sexes (Table S1).

We used heterosis as a measure of the relative share of the population's mutation load that is captured within each of our strains. Heterosis among inbred strains of a population should predominantly owe to the same type of genetic variation that is often expected to constitute a population's mutation load—rare, partially recessive deleterious alleles (Haldane 1927; Lande 1975; Houle et al. 1997; Lynch et al. 1999; Zhang et al. 2004; Charlesworth and Willis 2009). This is particularly likely in the case of our study population, as its genetic variance in fitness is characteristic of that underlain by rare, partially recessive deleterious alleles: fitness in the inbred state exhibits a substantially lower mean and greater variance relative to the outbred state (Robertson 1952; Houle et al. 1997; Kelly 1999; Charlesworth and Hughes 2000; Kelly and Tourtellot 2006; Table 1; Fig. 1A). Further, although this synthetic diallel population (Grieshop and Arnqvist 2018) as well as its wild‐caught origins (Berger et al. 2014, 2016a) bear the hallmarks of fitness variance maintained by sexually antagonistic balancing selection, this sexually antagonistic genetic variation apparently plays little or no role in determining the magnitude of heterosis in crosses among inbred strains, as the magnitude of heterosis experienced on average among strains is nearly identical between the sexes (Fig. 1B). In accordance, a previous estimate of dominance variance in this population, which is based to a large extent on heterosis (Hayman 1954; Lynch and Walsh 1998; Lenarcic et al. 2012; Maurizio et al. 2018; Shorter et al. 2019), was likewise found to describe sexually concordant fitness effects (Grieshop and Arnqvist 2018). Reciprocally, the sex‐reversed dominance effects (Kidwell et al. 1977; Fry 2010; Barson et al. 2015; Spencer and Priest 2016; Connallon and Chenoweth 2019) that were previously identified in this population (Grieshop and Arnqvist 2018) were detected via methods that are not based on heterosis. Thus, there is very little, if any, scope for this population's genetic variance in heterosis to be attributable to factors other than rare, partially recessive deleterious alleles, and we are confident that the most relevant form of the “segregation load” (see *Introduction*) that could possibly confound this interpretation—that is, sexually antagonistic genetic variation—is absent from our estimates of sex‐/strains‐specific heterosis (further discussion in Appendix C). The genetic covariance between those heterosis estimates and sex‐specific outbred fitness therefore indicates how selection would act to purge the alleles that make up this population's mutation load.

Our findings are pertinent to the longstanding question of why sexual reproduction is so prevalent in nature. Because, all else equal, the production of sons would halve the exponential growth rate of a sexual female's lineage relative to an asexual competitor (Maynard Smith 1971; Lehtonen et al. 2012; Gibson et al. 2017), there must be some mechanism(s) that compensate for the twofold cost of sex. One explanation is that the efficacy of selection against the mutation load on a population's offspring production is greater in males relative to females, which would allow that load to be purged without the demographic costs that would ensue given that same strength of selection acting on the population's females (Manning 1984; Kodric‐Brown and Brown 1987; Agrawal 2001; Siller 2001; Whitlock 2002; Lorch et al. 2003; Whitlock and Agrawal 2009). Our most relevant estimate of the relative extent to which our population's mutation load is purged via selection in males versus females is the fold difference between $\beta_{a_{M}}^{{}^{\prime \prime }}$ and $\beta_{a_{F}}^{{}^{\prime \prime }}$. Although these estimates were not statistically significantly different from one another, the male estimate was highly significant and the female estimate clearly was not (as was the case for $\beta_{a_{M}}^{\prime }$ and $\beta_{a_{M}}^{\prime }$; Table S1). As a rough guide, selection against rare, partially recessive deleterious mutations was estimated to be 3.7× greater in males than in females, in accordance with previous, more general, estimates of sex‐specific selection in this species: approximately three times stronger in males for selection against induced mutations (Grieshop et al. 2016) and two to four times stronger for males in outbred populations (Fritzsche and Arnqvist 2013; Martinossi‐Allibert et al. 2020). We note that the fold difference of male bias in selection that is needed for the production of males to yield a net benefit to females/populations does, however, depend on the genome‐wide deleterious mutation rate, as well as other genetic, demographic, and ecological factors (Agrawal 2001; Siller 2001; Agrawal and Whitlock 2012). This includes any costs brought on by sexual conflict (Whitlock and Agrawal 2009; Lehtonen et al. 2012; Burke and Bonduriansky 2017). Indeed, both intra‐ and interlocus sexual conflicts—that is, sexually antagonistic selection on sex‐homologous and sex‐heterologous traits, respectively—impose costs to our population's offspring production (Berger et al. 2016a), which likely drives the cost of sex to be greater than twofold. Nevertheless, at the very least, our findings indicate that the ability of selection to purge the population's mutation load is detectable via males, but absent in females, representing a striking difference between the sexes that may partially compensate for the cost of sex.

### MECHANISTIC UNDERSTANDING

One explanation for why male fitness exhibits greater sensitivity to mutation load is that the fitness consequences of genetic variation in traits under selection are greater (i.e., phenotypic selection is stronger) in males, and/or phenotypic variance in fitness‐related traits is more sensitive to mutational input in males (Rowe and Houle 1996). That is, rare partially recessive deleterious mutations may not manifest in female fitness components strongly enough, and/or those female fitness components may not vary enough, to expose those deleterious alleles to selection as readily as in males. Indeed, outbred males exhibited 1.24× the genetic variance in fitness relative to outbred females (Table 1), and males appear to have suffered moderately greater detriments than females from having their partially recessive deleterious alleles revealed by inbreeding/homozygosity (see inbred points above the *y* = *x* line in Figs. 1A and S5). Although the present data do not allow us to distinguish between whether male fitness variance is greater because phenotypic selection is stronger in males or because phenotypic traits under selection are more variable in males, these broader characteristics of our population may hold across other animal taxa. Laboratory estimates from insects based on inbreeding depression (Mallet and Chippindale 2011), mutation accumulation (Mallet et al. 2011, 2012; Sharp and Agrawal 2013), and induced mutations (Sharp and Agrawal 2008; Almbro and Simmons 2014; Grieshop et al. 2016) indirectly suggest that males are indeed more sensitive to mutation load than females. Further, meta‐analyses show that the opportunity for, and the strength of, selection is generally greater in males than females (e.g., Janicke et al. 2016; Singh and Punzalan 2018).

Quantitative genetic studies of sex‐biased genes (genes with sexually dimorphic expression) provide further mechanistic insight to our findings. Male fitness components in *Drosophila melanogaster* are, at least to some extent, determined by the expression levels of genes that typically show male‐biased expression (Dean et al. 2018). Further, the expression levels of male‐biased genes of *D. serrata* exhibit greater broad‐sense heritability than those of female‐biased genes (Allen et al. 2018), suggesting that selection could act more efficiently to purge deleterious alleles from any sites that affect the expression levels of male‐biased genes. As for how this might affect female fitness, that same study found higher intersexual genetic correlations ($r_{MF}$) for expression in male‐biased versus female‐biased genes (Allen et al. 2018). High $r_{MF}$ for gene expression or other traits is often interpreted as genetic constraints to sexual dimorphism, possibly imposing sexually antagonistic fitness consequences (Bonduriansky and Chenoweth 2009; Cox and Calsbeek 2009; van Doorn 2009; Connallon et al. 2010; Stewart et al. 2010; Griffin et al. 2013; Ingleby et al. 2014; McGlothlin et al. 2019; Kollar et al. 2021). However, it is certainly still possible for such male‐biased genes to have sexually concordant fitness effects, a core assumption of the “condition dependence” theory for sexually selected traits (Rowe and Houle 1996). Indeed, although mutations in *D. melanogaster*’s male‐ and female‐biased genes had greater detriments to male and female fitness components, respectively, the direction of these effects nevertheless tends to be sexually concordant—that is, detrimental in both sexes (Connallon and Clark 2011). Of particular relevance to the present study, the large majority of male‐biased genes in *C. maculatus* that are expressed in females are actually upregulated in females after mating (Immonen et al. 2017). Thus, much of the mutation load on female reproduction in *C. maculatus* could manifest via the expression of male‐biased genes, which the present findings show would be purged more effectively via males. Accordingly, *C. maculatus* male‐biased genes show a clear pattern of purifying selection that is not seen in female‐biased genes (Sayadi et al. 2019).

Although our findings do not offer direct support of “good genes” sexual selection, they do represent evidence of the prerequisite conditions for that process (Zahavi 1975; Lande 1981; Hamilton and Zuk 1982; Grafen 1990; Kirkpatrick and Ryan 1991; Andersson 1994; Kirkpatrick 1996; Kirkpatrick and Barton 1997; Martinossi‐Allibert et al. 2019a). Empirical evidence for good genes sexual selection remains scant (Prokop et al. 2012). For good genes sexual selection to work, female choosiness should covary with the “genetic quality” of their mates (i.e., males’ breeding values for fitness; Hunt et al. 2004). Further, that genetic quality should be passed on to both sons and daughters. Lastly, “genic capture” (i.e., polygenic mutation‐selection balance; Rowe and Houle 1996; Tomkins et al. 2004) should prevent variation in genetic quality from being depleted. Although our fitness estimates are not a measure of female choice, our findings are consistent with male breeding values for fitness (i.e., genetic quality) reflecting polygenic deleterious mutational variation. Although male mating success in this species does seem more to do with male competition than female choice (Savalli and Fox 1999), the kicking behavior that females exhibit may still serve as a baseline level of resistance that enables females to choose the males that are capable of overcoming it (Maklakov and Arnqvist 2009). Further, postcopulatory cryptic female choice (Thornhill 1983; Eberhard 1996; Pitnick et al. 2009; Arnqvist 2014) may comprise a large fraction of male fitness variance in this species (Hotzy et al. 2012; Fritzsche and Arnqvist 2013; Bayram et al. 2019), although there is little evidence for “good genes” effects operating in this context (Bilde et al. 2008, 2009). Thus, although only direct selection on female choice has been demonstrated in this system (Maklakov and Arnqvist 2009), the current findings show that the prerequisite genetic architecture is present for indirect “good genes” effects to act in conjunction with direct selection (Kirkpatrick and Barton 1997).

For selection via males to yield net benefits to females/populations, thereby contributing to the maintenance of sexual reproduction and female trait preferences via “good genes,” selection in the long run should necessarily act to purge unconditionally deleterious alleles. However, the ability to detect this process in the standing genetic variation or short‐term evolutionary outcomes may be overshadowed by genetic variation whose effects are conditional upon sex (Whitlock and Agrawal 2009). Our study population is known to originally harbor sexually antagonistic standing genetic variance in fitness (Berger et al. 2014, 2016a), and as discussed above, the synthetic diallel population analyzed here apparently still does consist of some sexually antagonistic genetic variation (Grieshop and Arnqvist 2018). The present findings are thus a testament to the fact that selection in males against unconditionally deleterious alleles is still detectable and able to promote female offspring production despite the male‐imposed detriments of sexually antagonistic genetic variation.

## AUTHOR CONTRIBUTIONS

KG, DB, and GA conceived of and designed the experiment. KG conducted the experiment. KG, DB, and PLM conducted the statistical analyses. KG wrote the first draft of the manuscript. All authors contributed to editing the manuscript.

## DATA ARCHIVING

The raw data, R code, a dependency, and the model output files—which together enable all analyses, procedures, figures, and tables to be reproduced—have been submitted for review under the reported settings and are available at Dryad DOI https://doi.org/10.5061/dryad.p5hqbzkpn.

## Supporting information




**Table S1**. Comparison and rationale for using opposite‐sex heterosis for point estimates and sex‐averaged heterosis for sex‐differences.
**Fig. S1**. Lome population history and experimental design.
**Fig. S2**. The raw‐means *r*_MF_ of the isofemale lines.
**Fig. S3**. Results the Gelman‐Rubin analysis, demonstrating good mixing of four independent MCMC chains.
**Fig. S4**. Outbred (*o*) breeding values from Fig. 1A shaded by sex‐averaged heterosis.
**Fig. S5**. Inbred (*i*) breeding values from Fig. 1A shaded by sex‐averaged heterosis.
**Fig. S6**. Resampled point estimates and 95% credibility intervals of $\beta_{a}^{\prime }$ for males ($\beta_{a_{M}}^{\prime }$) and females ($\beta_{a_{F}}^{\prime }$).

## APPENDIX A

**Table A1:** Frequently used symbols from the main text

Symbol	Meaning
$\beta_{a_{F}}^{\prime }$	Standardized estimate of selection in females against deleterious effects revealed in male heterosis (eq. 3b)
$\beta_{a_{M}}^{\prime }$	Standardized estimate of selection in males against deleterious effects revealed in female heterosis (eq. 3a)
$\beta_{a_{F}}^{{}^{\prime \prime }}$	Standardized estimate of selection in females against deleterious effects revealed in sex‐averaged heterosis
$\beta_{a_{M}}^{{}^{\prime \prime }}$	Standardized estimate of selection in males against deleterious effects revealed in sex‐averaged heterosis
$i_{F}$	Female inbred fitness
$i_{M}$	Male inbred fitness
$o_{F}$	Female outbred fitness
$o_{M}$	Male outbred fitness
o−i	Heterosis
$r_{o_{F},\;\;o_{M}-i_{M}}$	Genetic correlation between outbred female fitness and male heterosis (eq. 5b)
$r_{o_{M},\;\;o_{F}-i_{F}}$	Genetic correlation between outbred male fitness and female heterosis (eq. 5a)
COV(x,y)	Genetic covariance between x and y
SD(x)	Genetic standard deviation in x
V(x)	Genetic variance in x

**Table A2:** H matrix (with CIs and residual variances) estimated for relative *Y*‐adjusted fitness (i.e., relative fitness after removing parental effects; see *Methods* and Appendix B), displaying genetic variances (and residual variances) on the diagonal, their covariances in the lower triangle, and their Pearson's correlation coefficients (*r*) in the upper triangle. Covariances and correlations with 95% credibility intervals excluding zero are bolded

	*i_F_*	*o_F_*	*i_M_*	*o_M_*
*i_F_*	0.0109 (0.004, 0.021) (0.0257)	0.35	**0.82**	0.48
*o_F_*	0.0011	0.0015 (<0.001, 0.003) (0.0149)	0.16	−0.02
*i_M_*	**0.0106**	0.0002	0.0195 (0.006, 0.038) (0.0857)	**0.70**
*o_M_*	0.0017	−1.8 × 10^–5^	0.0030	0.0019 (<0.001, 0.004) (0.0836)

## APPENDIX B

### Regarding the role of parental effects and sex chromosomes

#### Rationale

There is the potential that nongenetic parental effects of each strain could partly confound our additive genetic interpretation of the *o_M_* and *o_F_* estimates. That is, the *o_M_* and *o_F_* estimates of each point on Figure 1C stem from replicate observations of the same genetic crosses, meaning that in one extreme limit, differences among them could be attributable to the inheritance/transfer of phenotypic condition from those same recurrent strains to their F_1_ offspring rather than the inheritance of the genetic makeup of those strains, per se. Such “condition transfer” via parent‐of‐origin effects (e.g., epigenetics) may be a common adaptive feature of many organisms (Bonduriansky and Crean 2018). Further, genes with male‐biased gene expression, which we discuss as possibly mediating our findings (see MECHANISTIC UNDERSTANDING), may be likewise particularly relevant to condition transfer via parent‐of‐origin effects, as they have been shown to exhibit elevated condition‐dependent expression in *Drosophila melanogaster* (Wyman et al. 2010). In addition to nongenetic parental effects, our main finding of sex differences in the relationship between heterosis and outbred breeding values for fitness could be partly attributable to the mutation load carried only by males on the Y‐chromosome, and/or that revealed only in males on their unmasked hemizygous X‐chromosome, whereas X‐chromosome heterozygosity might mask these effects in females.

We sought to address these potential concerns. Diallel data lend themselves particularly well to identifying these effects via contrasts of reciprocal full siblings—pairs of crosses that are autosomally identical but have inherited their sex‐chromosomes, mitochondria, cytoplasm, and other epigenetic information from opposite strains (e.g., the F_1_ offspring of a strain‐1 father and strain‐2 mother are reciprocal full siblings with the F_1_s of a strain‐2 father and strain‐1 mother; see Fig. S1B). These parent‐of‐origin effects were identified via diallel variance partitioning (after Shorter et al. 2019) and removed from the fitness data, yielding a new “Y‐adjusted” fitness variable that was subject to the same analyses reported in the *Methods*.

#### Methods

To estimate and remove the contribution of strain‐specific parental sex effects (m and $\phi^{m}$) and asymmetric epistatic effects (w and $\phi^{w}$) from our fitness phenotype, y, resulting in $y^{\mathrm{adjusted}}$, we implemented an updated version of the linear mixed model previously used in Shorter et al. (2019), using the R software package MCMCglmm (Hadfield 2010). We used the BayesDiallel “fulls” (full, sexed, “BSabmvw”) model to estimate the contribution of parental strains and their various effects as described in Lenarcic et al. (2012), where for an individual *i* the phenotype is modeled as

$$\;y_{i}=\mu^{d}\;+\alpha_{\mathrm{block}\left[i\right]}+d^{T}\beta +\varepsilon_{i},$$

with $\varepsilon_{i}∼N\left(0,\;\sigma^{2}\right)$. Here, the overall mean and block effects are modeled by $\mu^{d}$ and $\alpha_{\mathrm{block}}$, respectively. For dam *j*, sire *k*, and sex *s*, with indicator functions ψ, the diallel effects, $d^{T}\beta$, are estimated by

$$\begin{array}{ccc} {d^{T}}_{\left(jks\right)}\beta & = & a_{j}+a_{k}+m_{j}-m_{k}+\psi_{\left\{j\;=\;k\right\}}\left(\beta_{\mathrm{inbred}}+b_{j}\right)\\ & & +\;\psi_{\left\{j\neq k\right\}}\left(v_{jk}^{d}+\psi_{j<k}\cdot w_{jk}\right)\\ & & +\;\psi_{\left\{s\;=\;\mathrm{female}\right\}}\cdot \left[\phi +\phi_{j}^{a}+\;\phi_{k}^{a}+\phi_{j}^{m}\right.\\ & & \left.-\;\phi_{k}^{m}+\psi_{\left\{j\;=\;k\right\}}\left(\phi_{\mathrm{inbred}}+\phi_{j}^{b}\right)\right.\\ & & \left.+\;\psi_{\left\{j\neq k\right\}}\left(\phi_{jk}^{v}+\psi_{j<k}\cdot \phi_{jk}^{w}\right)\right], \end{array}$$

where for all *j* strains, the strain‐specific effects are modeled marginally for additive effects as $a_{1},\;a_{2},\;\ldots ,\;a_{J}∼N\left(0,\tau_{a}^{2}\right)$, parental sex effects as $m_{1},\;m_{2},\;\ldots ,\;m_{J}∼N\left(0,\tau_{m}^{2}\right)$, inbred effects $b_{1},\;b_{2},\ldots ,\;b_{J}∼N\left(0,\tau_{b}^{2}\right)$, and similarly for sex‐specific versions of these effects classes ($\phi^{a},\;\phi^{m},\phi^{b}$). The strainpair‐specific effects for all unique *j*‐by‐*k* noninbred combinations are modeled similarly for symmetric epistatic ($v^{d}$), asymmetric epistatic (w), and sex‐specific versions ($\phi^{v}\;\mathrm{and}\;\phi^{w}$, respectively), for example, as marginally $w_{jk}∼N\left(0,\tau_{w}^{2}\right)$ for asymmetric epistatic effects.

The priors for the five fixed effects ($\mu^{d},\;\alpha_{\mathrm{block}},\;\beta_{\mathrm{inbred}},\;\phi \;\left[\mathrm{overall}\;\mathrm{female}\right]$, and $\phi_{\mathrm{inbred}}$) are each set to a vague normal distribution, $\mathrm{fixed}\;\mathrm{effect}\;∼\;N(0,10^{3})$, and the priors for variance of the residuals ($\sigma^{2}$) and for the variance for each class of effects ($\tau_{a}^{2},\;\tau_{m}^{2},\tau_{b}^{2},\tau_{v}^{2},\tau_{w}^{2}$, and sex‐specific versions) are set as a weakly informative Inverse‐Wishart with V = 2, and nu = 0.002, equivalent to an inverse gamma prior with shape and scale of 0.001. The estimates are based on models that were run for 17,000 iterations after 2000 iterations of burn‐in.

After obtaining stable estimates, for example, $\hat{m}$ for parental effects, for all strains and strain pairs, the original fitness phenotypes are adjusted using the following relationship for each *j*, *k*, and *s*

$$\;y_{\;jk,\;s\;=\;\mathrm{male}}^{\mathrm{adjusted}}=y_{jks}\;-\left(\hat{m_{j}}-\hat{m_{k}}\right)-\hat{w_{jk}}$$

$$\;y_{\;jk,\;s\;=\;\mathrm{female}}^{\mathrm{adjusted}}=y_{jks}\;-\left(\hat{m_{j}}-\hat{m_{k}}\right)-\hat{w_{jk}}-\left(\hat{\phi_{j}^{m}}-\hat{\phi_{k}^{m}}\right)-\hat{\phi_{jk}^{w}}.$$

These adjusted *Y* fitness values are then modeled in the same way as the original fitness values in the main text (see *Methods*).

#### Results

The results we obtained using the Y_adjusted fitness values were highly consistent with those reported in the main text. The model fits (see eq. 2, *Methods*) before and after removing the parental effects were negligibly different (DIC = −1219.80 and −1222.275, respectively). The standardized H matrix estimated after removing the parental effects from the data (Table A1) was qualitatively similar to before (Table 1). Resampling this model revealed that heterosis in males and females was still highly positively correlated ($r_{o_{M}-i_{M},\;\;o_{F}-i_{F}}$ = 0.84 [0.67, 0.96], *P* < 0.001). Likewise, male outbred breeding values were highly negatively correlated with heterosis in females ($r_{o_{M},\;\;o_{F}-i_{F}}$ = −0.53 [−0.84, −0.14], *P* = 0.014), whereas female outbred breeding values were not correlated to heterosis in males ($r_{o_{F},\;\;o_{M}-i_{M}}$ = −0.09 [−0.44, 0.25], *P* = 0.674). Thus, our analysis suggests that nongenetic parental effects, sex‐chromosome effects, and their epistatic interactions with autosomal genetic variation do not contribute to our main findings. For any parental effects variance to still be present in the *Y*‐adjusted fitness variable, and hence still clouding our interpretation, the pattern of strain‐specific nongenetic inheritance would need to be identical between reciprocal full‐sibling contrasts (as well as between the sex‐specific, i.e., son‐daughter, contrasts of those reciprocal full‐sibling differences). We argue that this is highly unlikely considering that parental effect variance manifests in fathers and mothers via fundamentally different and highly asymmetric pathways (e.g., cytoplasmic, mitochondrial, and sex‐chromosome inheritance).

## APPENDIX C

### Regarding sexually antagonistic variants and heterosis

In an extreme case, in principle, the present synthetic diallel population may be largely fixed throughout all 16 inbred strains for female‐benefit sexually antagonistic alleles at many genetic loci due to excess lineage extinction among inbred lines that originated from isofemale lines that were enriched for male‐benefit/female‐detriment sexually antagonistic genetic variants (Grieshop et al. 2017). Yet, some remaining sexually antagonistic polymorphisms demonstrably still segregate among the inbred strains (Grieshop and Arnqvist 2018). Under one scenario, some strains could be completely fixed for the female‐benefit alleles across all sexually antagonistic loci, while other strains may be fixed for most, but not all, female benefit alleles, and thus fixed for male‐benefit alleles at the remining sites. Under diminishing returns epistasis (Whitlock et al. 1995; Martin et al. 2007; Berger and Postma 2014), which is theoretically predicted among polymorphic sexually antagonistic sites (Arnqvist et al. 2014), strains fixed for female‐benefit alleles throughout all sexually antagonistic loci would tend to have relatively low male fitness (*o_M_*) and relatively high measures of male heterosis (*o_M_*–*i_M_*) owing to the recruitment of male‐benefit alleles (upon outcrossing with strains that are fixed for some male‐benefit alleles) in an otherwise male‐detriment genetic background. By contrast, strains that have some male‐benefit alleles fixed would have relatively high male fitness and relatively low measures of male heterosis, as they have less to gain than strains that are fixed for female‐benefit alleles across all sexually antagonistic loci (Arnqvist et al. 2014). Female fitness and heterosis would be relatively unaffected in this context because they are either completely or mostly saturated for alleles that benefit their fitness, making the recruitment of additional female‐benefit alleles in an otherwise female‐benefit background relatively ineffectual (Arnqvist et al. 2014). At first glance, this explanation seems to match our results in that fitness would be negatively correlated to heterosis in males but not females. However, our main finding is specifically between male fitness (*o_M_*) and *female* heterosis (*o_F_*–*i_F_*) (Fig. 1C). Further, *o_M_*–*i_M_* ≈ *o_F_*–*i_F_* (Fig. 1B). Neither of those findings are compatible with an explanation based on sexually antagonistic genetic variation (and sexually antagonistic diminishing returns epistasis) underlying heterosis.

## References

[evl3239-bib-0001] Agrawal, A. F. 2001. Sexual selection and the maintenance of sexual reproduction. Nature 411: 692–695. 11395771 10.1038/35079590

[evl3239-bib-0002] Agrawal, A. F. , and M. C. Whitlock 2012. Mutation load: the fitness of individuals in populations where deleterious alleles are abundant. Annu Rev Ecol Evol Syst 43:115–135.

[evl3239-bib-0003] Allen, S. L. , R. Bonduriansky , and S. F. Chenoweth 2018. Genetic constraints on microevolutionary divergence of sex‐biased gene expression. Philos Trans R Soc Lond B Biol Sci 373:20170427. 30150225 10.1098/rstb.2017.0427 PMC6125734

[evl3239-bib-0004] Almbro, M. , and L. W. Simmons 2014. Sexual selection can remove an experimentally induced mutation load. Evolution 68:295–300. 24372608 10.1111/evo.12238

[evl3239-bib-0005] Andersson, M. 1994. Sexual selection. Princeton Univ. Press, Princeton, NJ.

[evl3239-bib-0160] Arnqvist, G., N. Vellnow , and L. Rowe . 2014. The effect of epistasis on sexually antagonistic genetic variation. Proc. Biol. Sci. 281:20140489. 24870040 10.1098/rspb.2014.0489 PMC4071542

[evl3239-bib-0006] Arnqvist, G. 2014. Cryptic female choice. In: The evolution of insect mating systems, {eds. Shuker, D.M. and Simmons, L.W. }. Oxford Univ. Press, USA, Cary, NC, pp.204–220.

[evl3239-bib-0007] Barson, N. J. , T. Aykanat , K. Hindar , M. Baranski , G. H. Bolstad , P. Fiske , *et al*. 2015. Sex‐dependent dominance at a single locus maintains variation in age at maturity in salmon. Nature 528:405–408. 26536110 10.1038/nature16062

[evl3239-bib-0008] Barton, N. H. , and P. D. Keightley 2002. Understanding quantitative genetic variation. Nat Rev Genet 3:11–21. 11823787 10.1038/nrg700

[evl3239-bib-0009] Bayram, H. , A. Sayadi , E. Immonen , and G. Arnqvist 2019. Identification of novel ejaculate proteins in a seed beetle and division of labour across male accessory reproductive glands. Insect Biochem Mol Biol 104:50–57. 30529580 10.1016/j.ibmb.2018.12.002

[evl3239-bib-0010] Berger, D. , and E. Postma 2014. Biased estimates of diminishing‐returns epistasis? Empirical evidence revisited. Genetics 198:1417–1420. 25313131 10.1534/genetics.114.169870 PMC4256761

[evl3239-bib-0011] Berger, D. , K. Grieshop , M. I. Lind , J. Goenaga , A. A. Maklakov , and G. Arnqvist 2014. Intralocus sexual conflict and environmental stress. Evolution 68:2184–2196. 24766035 10.1111/evo.12439

[evl3239-bib-0012] Berger, D. , I. Martinossi‐Allibert , K. Grieshop , M. I. Lind , A. A. Maklakov , and G. Arnqvist 2016a. Intralocus sexual conflict and the tragedy of the commons in seed beetles. Am Nat 188:E98–E112. 27622882 10.1086/687963

[evl3239-bib-0013] Berger, D. , T. You , M. R. Minano , K. Grieshop , M. I. Lind , G. Arnqvist , et al. 2016b. Sexually antagonistic selection on genetic variation underlying both male and female same‐sex sexual behavior. BMC Evol Biol 16:88. 27175796 10.1186/s12862-016-0658-4 PMC4866275

[evl3239-bib-0014] Bilde, T. , U. Friberg , A. A. Maklakov , J. D. Fry , and G. Arnqvist 2008. The genetic architecture of fitness in a seed beetle: assessing the potential for indirect genetic benefits of female choice. BMC Evol Biol 8:1–11. 18950531 10.1186/1471-2148-8-295 PMC2596129

[evl3239-bib-0015] Bilde, T. , A. Foged , N. Schilling , and G. Arnqvist 2009. Postmating sexual selection favors males that sire offspring with low fitness. Science 324:1705–1706. 19556506 10.1126/science.1171675

[evl3239-bib-0016] Bonduriansky, R. , and S. F. Chenoweth 2009. Intralocus sexual conflict. Trends Ecol Evol 24:280–288. 19307043 10.1016/j.tree.2008.12.005

[evl3239-bib-0161] Bonduriansky, R. , and A. J. Crean . 2018. What are parental condition‐transfer effects and how can they be detected? Methods Ecol. Evol. 9:450–456.

[evl3239-bib-0017] Burke, N. W. , and R. Bonduriansky 2017. Sexual conflict, facultative asexuality, and the true paradox of sex. Trends Ecol Evol 32:646–652. 28651895 10.1016/j.tree.2017.06.002

[evl3239-bib-0018] Buzatto, B. A. , and H. L. Clark 2020. Selection for male weapons boosts female fecundity, eliminating sexual conflict in the bulb mite. Sci Rep 10:1–7. 32047190 10.1038/s41598-020-59254-2 PMC7012872

[evl3239-bib-0019] Cally, J. G. , D. Stuart‐Fox , and L. Holman 2019. Meta‐analytic evidence that sexual selection improves population fitness. Nat comm 10:1–10. 10.1038/s41467-019-10074-7 PMC6494874 31043615

[evl3239-bib-0020] Charlesworth, B. 2015. Causes of natural variation in fitness: evidence from studies of *Drosophila* populations. Proc Natl Acad Sci USA 112:1662–1669. 25572964 10.1073/pnas.1423275112 PMC4330759

[evl3239-bib-0021] Charlesworth, B. , and D. Charlesworth 1999. The genetic basis of inbreeding depression. Genet Res 74:329–340. 10689809 10.1017/s0016672399004152

[evl3239-bib-0022] ———. 2010. Elements of evolutionary genetics. Roberts and Company Publishers, Greenwood Village, CO.

[evl3239-bib-0023] Charlesworth, B. , and K. A. Hughes 2000. The maintenance of genetic variation in life‐history traits. In: (Evolutionary genetics), {eds. Singh, R.S. , Krimbas, C.B. }. Cambridge Univ. Press, Cambridge, U.K., pp.369–392.

[evl3239-bib-0024] Charlesworth, B. , T. Miyo , and H. Borthwick 2007. Selection responses of means and inbreeding depression for female fecundity in *Drosophila melanogaster* suggest contributions from intermediate‐frequency alleles to quantitative trait variation. Genet Res 89:85–91. 17521472 10.1017/S001667230700866X

[evl3239-bib-0025] Charlesworth, D. , and B. Charlesworth 1987. Inbreeding depression and its evolutionary consequences. Annu Rev Ecol Evol Syst 18:237–268.

[evl3239-bib-0026] Charlesworth, D. , and J. H. Willis 2009. The genetics of inbreeding depression. Nat Rev Genet 10:783–796. 19834483 10.1038/nrg2664

[evl3239-bib-0027] Cheng, C. , and D. Houle 2020. Predicting multivariate responses of sexual dimorphism to direct and indirect selection. Am Nat 196:391–405. 32970462 10.1086/710353

[evl3239-bib-0028] Chenoweth, S. F. , N. C. Appleton , S. L. Allen , and H. D. Rundle 2015. Genomic evidence that sexual selection impedes adaptation to a novel environment. Curr Biol 25:1860–1866. 26119752 10.1016/j.cub.2015.05.034

[evl3239-bib-0029] Chippindale, A. K. , J. R. Gibson , and W. R. Rice 2001. Negative genetic correlation for adult fitness between sexes reveals ontogenetic conflict in *Drosophila* . Proc Natl Acad Sci USA 98:1671–1675. 11172009 10.1073/pnas.041378098 PMC29315

[evl3239-bib-0030] Connallon, T. , and S. F. Chenoweth 2019. Dominance reversals and the maintenance of genetic variation for fitness. PLoS Biol 17:3000118. 10.1371/journal.pbio.3000118 PMC6368311 30695026

[evl3239-bib-0031] Connallon, T. , and A. G. Clark 2011. Association between sex‐biased gene expression and mutations with sex‐specific phenotypic consequences in *Drosophila* . Genome Biol Evol 3:151–155. 21292631 10.1093/gbe/evr004 PMC3048362

[evl3239-bib-0032] ———. 2012. A general population genetic framework for antagonistic selection that accounts for demography and recurrent mutation. Genetics 190:1477–1489. 22298707 10.1534/genetics.111.137117 PMC3316657

[evl3239-bib-0035] Connallon, T. , R. M. Cox , and R. Calsbeek 2010. Fitness consequences of sex‐specific selection. Evolution 64:1671–1682. 20050912 10.1111/j.1558-5646.2009.00934.x

[evl3239-bib-0036] Cox, R. M. , and R. Calsbeek 2009. Sexually antagonistic selection, sexual dimorphism, and the resolution of intralocus sexual conflict. Am Nat 173:176–187. 19138156 10.1086/595841

[evl3239-bib-0037] Crow, J. F. 1958. Some possibilities for measuring selection intensities in man. Hum Biol 30:1–13. 13513111

[evl3239-bib-0038] Dean, R. , C. Hammer , V. Higham , and D. K. Dowling 2018. Masculinization of gene expression is associated with male quality in *Drosophila melanogaster* . Evolution 72:2736–2748. 30382578 10.1111/evo.13618

[evl3239-bib-0040] Dugand, R. J. , W. J. Kennington , and J. L. Tomkins 2018. Evolutionary divergence in competitive mating success through female mating bias for good genes. Sci Adv 4:eaaq0369. 29806021 10.1126/sciadv.aaq0369 PMC5966190

[evl3239-bib-0041] ———. 2019a. Evaluating the genetic architecture of quantitative traits via selection followed by inbreeding. Heredity 123:407–418. 30967644 10.1038/s41437-019-0219-x PMC6781166

[evl3239-bib-0042] Dugand, R. J. , J. L. Tomkins , and W. J. Kennington 2019b. Molecular evidence supports a genic capture resolution of the lek paradox. Nat Comm 10:1–8. 10.1038/s41467-019-09371-y PMC6433924 30911052

[evl3239-bib-0043] Eberhard, W. 1996. Female control: sexual selection by cryptic female choice. Princeton Univ. Press, Princeton, NJ.

[evl3239-bib-0044] Falconer, D. S. , and T. F. C. Mackay 1996. Introduction to quantitative genetics. 2nd ed. Longman Group, Essex, U.K.

[evl3239-bib-0046] Firman, R. C. , and L. W. Simmons 2010. Experimental evolution of sperm quality via postcopulatory sexual selection in house mice. Evolution 64:1245–1256. 19922447 10.1111/j.1558-5646.2009.00894.x

[evl3239-bib-0047] ———. 2012. Male house mice evolving with post‐copulatory sexual selection sire embryos with increased viability. Ecol Lett 15:42–46. 22011211 10.1111/j.1461-0248.2011.01706.x

[evl3239-bib-0048] Fritzsche, K. , and G. Arnqvist 2013. Homage to Bateman: sex roles predict sex differences in sexual selection. Evolution 67:1926–1936. 23815650 10.1111/evo.12086

[evl3239-bib-0049] Fry, J. D. 2010. The genomic location of sexually antagonistic variation: some cautionary comments. Evolution 64:1510–1516. 19922443 10.1111/j.1558-5646.2009.00898.x PMC3654548

[evl3239-bib-0050] Gelman, A. , and D. B. Rubin 1992. Inference from iterative simulation using multiple sequences. Stat Sci 7:457–472.

[evl3239-bib-0051] Gibson, A. K. , L. F. Delph , and C. M. Lively 2017. The two‐fold cost of sex: experimental evidence from a natural system. Evol Lett 1:6–15. 30233811 10.1002/evl3.1 PMC6089407

[evl3239-bib-0052] Gosden, T. P. , and S. F. Chenoweth 2014. The evolutionary stability of cross‐sex, cross‐trait genetic covariances. Evolution 68:1687–1697. 24620712 10.1111/evo.12398

[evl3239-bib-0053] Grafen, A. 1990. Biological signals as handicaps. J Theor Biol 144:517–546. 2402153 10.1016/s0022-5193(05)80088-8

[evl3239-bib-0054] Grieshop, K. , and G. Arnqvist 2018. Sex‐specific dominance reversal of genetic variation for fitness. PLoS Biol 16:2006810. 10.1371/journal.pbio.2006810 PMC6303075 30533008

[evl3239-bib-0163] Grieshop, K. , and G. Arnqvist . 2018. Sex‐specific dominance reversal of genetic variation for fitness. PLoS Biol. 16:e2006810. 30533008 10.1371/journal.pbio.2006810 PMC6303075

[evl3239-bib-0055] Grieshop, K. , J. Stångberg , I. Martinossi‐Allibert , G. Arnqvist , and D. Berger 2016. Strong sexual selection in males against a mutation load that reduces offspring production in seed beetles. J Evol Biol 29:1201–1210. 26991346 10.1111/jeb.12862

[evl3239-bib-0056] Grieshop, K. , D. Berger , and G. Arnqvist 2017. Male‐benefit sexually antagonistic genotypes show elevated vulnerability to inbreeding. BMC Evol Biol 17:134. 28606137 10.1186/s12862-017-0981-4 PMC5469140

[evl3239-bib-0057] Griffin, R. M. , R. Dean , J. L. Grace , P. Rydén , and U. Friberg 2013. The shared genome is a pervasive constraint on the evolution of sex‐biased gene expression. Mol Biol Evol 30:2168–2176. 23813981 10.1093/molbev/mst121

[evl3239-bib-0058] Hadfield, J. 2012. MCMCglmm course notes. Available via http://cran.r‐project.org/web/packages/MCMCglmm/vignettes/CourseNotes.pdf.

[evl3239-bib-0059] Hadfield, J. D. 2010. MCMC methods for multi‐response generalized linear mixed models: the MCMCglmm R package. J Stat Softw 33:1–22. Available via http://www.jstatsoft.org/v33/i02/.20808728

[evl3239-bib-0060] Hadfield, J. D. , A. J. Wilson , D. Garant , B. C. Sheldon , and L. E. Kruuk 2010. The misuse of BLUP in ecology and evolution. Am Nat 175:116–125. 19922262 10.1086/648604

[evl3239-bib-0061] Haldane, J. B. S. 1927. A mathematical theory of natural and artificial selection, part V: selection and mutation. Proc Camb Philos Soc 23:838–844.

[evl3239-bib-0062] Hamilton, W. D. , and M. Zuk 1982. Heritable true fitness and bright birds: a role for parasites? Science 218:384–387. 7123238 10.1126/science.7123238

[evl3239-bib-0064] Hayman, B. I. 1954. The theory and analysis of diallel crosses. Genetics 39:789–809. 17247520 10.1093/genetics/39.6.789 PMC1209689

[evl3239-bib-0065] Hollis, B. , J. L. Fierst , and D. Houle 2009. Sexual selection accelerates the elimination of a deleterious mutant in *Drosophila melanogaster* . Evolution 63:324–333. 19154371 10.1111/j.1558-5646.2008.00551.x

[evl3239-bib-0066] Hotzy, C. , M. Polak , J. L. Rönn , and G. Arnqvist 2012. Phenotypic engineering unveils the function of genital morphology. Curr Biol 22:2258–2261. 23103188 10.1016/j.cub.2012.10.009

[evl3239-bib-0067] Houle, D. , K. A. Hughes , D. K. Hoffmaster , J. Ihara , S. Assimacopoulos , and B. Charlesworth 1994. The effects of spontaneous mutation on quantitative traits. I. Variances and covariances of life history traits. Genetics 138:773–785. 7851773 10.1093/genetics/138.3.773 PMC1206226

[evl3239-bib-0068] Houle, D. , K. A. Hughes , S. Assimacopoulos , and B. Charlesworth 1997. The effects of spontaneous mutation on quantitative traits. II. Dominance of mutations with effects on life‐history traits. Genet Res 70:27–34. 9369096 10.1017/s001667239700284x

[evl3239-bib-0070] Hunt, J. , L. F. Bussiere , M. D. Jennions , and R. Brooks 2004. What is genetic quality? Trends Ecol Evol 19:329–333. 16701279 10.1016/j.tree.2004.03.035

[evl3239-bib-0071] Ingleby, F. C. , P. Innocenti , H. D. Rundle , and E. H. Morrow 2014. Between‐sex genetic covariance constrains the evolution of sexual dimorphism in *Drosophila melanogaster* . J Evol Biol 27:1721–1732. 24893565 10.1111/jeb.12429

[evl3239-bib-0072] Immonen, E. , A. Sayadi , H. Bayram , and G. Arnqvist 2017. Mating changes sexually dimorphic gene expression in the seed beetle *Callosobruchus maculatus* . Genome Biol Evol 9:677–699. 28391318 10.1093/gbe/evx029 PMC5381559

[evl3239-bib-0073] Janicke, T. , I. K. Häderer , M. J. Lajeunesse , and N. Anthes 2016. Darwinian sex roles confirmed across the animal kingdom. Sci Adv 2:1500983. 10.1126/sciadv.1500983 PMC4758741 26933680

[evl3239-bib-0074] Katvala, M. , J. L. Rönn , and G. Arnqvist 2008. Correlated evolution between male ejaculate allocation and female remating behaviour in seed beetles (Bruchidae). J Evol Biol 21:471–479. 18205777 10.1111/j.1420-9101.2007.01494.x

[evl3239-bib-0075] Kelly, J. K. 1999. An experimental method for evaluating the contribution of deleterious mutations to quantitative trait variation. Genet Res 73:263–273. 10425922 10.1017/s0016672399003766

[evl3239-bib-0076] ———. 2003. Deleterious mutations and the genetic variance of male fitness components in *Mimulus guttatus* . Genetics 164:1071–1085. 12871916 10.1093/genetics/164.3.1071 PMC1462635

[evl3239-bib-0077] Kelly, J. K. , and M. K. Tourtellot 2006. The genetic analysis of family structured inbreeding depression studies. Heredity 97:346–354. 16896343 10.1038/sj.hdy.6800879

[evl3239-bib-0078] Kelly, J. K. , and J. H. Willis 2001. Deleterious mutations and genetic variation for flower size in *Mimulus guttatus* . Evolution 55:937–942. 11430654 10.1554/0014-3820(2001)055[0937:dmagvf]2.0.co;2

[evl3239-bib-0079] Kidwell, J. F. , M. T. Clegg , F. M. Stewart , and T. Prout 1977. Regions of stable equilibria for models of differential selection in the two sexes under random mating. Genetics 85:171–183. 838269 10.1093/genetics/85.1.171 PMC1213615

[evl3239-bib-0080] Kirkpatrick, M. 1996. Good genes and direct selection in the evolution of mating preferences. Evolution 50:2125–2140. 28565675 10.1111/j.1558-5646.1996.tb03603.x

[evl3239-bib-0081] Kirkpatrick, M. , and N. H. Barton 1997. The strength of indirect selection on female mating preferences. Proc Natl Acad Sci USA 94:1282–1286. 9037044 10.1073/pnas.94.4.1282 PMC19782

[evl3239-bib-0082] Kirkpatrick, M. , and M. J. Ryan 1991. The evolution of mating preferences and the paradox of the lek. Nature 350:33–38.

[evl3239-bib-0083] Kodric‐Brown, A. , and J. H. Brown 1987. Anisogamy, sexual selection, and the evolution and maintenance of sex. Evol Ecol 1:95–105.

[evl3239-bib-0157] Kollar, L.M. , Kiel, S. , James, A.J. , Carnley, C.T. , Scola, D.N. , Clark, T.N. , Khanal, T. , Rosentiel, T.N. , Gall, E.T. , Grieshop, K. , and McDaniel, S.F. 2021. The genetic architecture of sexual dimorphism in the moss Ceratodon purpureus. 288:20202908. Proceedings of the Royal Society B 288:20202908. 33715431 10.1098/rspb.2020.2908 PMC7944104

[evl3239-bib-0086] Kyogoku, D. , and T. Sota 2021. Sexual selection increased offspring production via evolution of male and female traits. J Evol Biol 34:501–511. 33314378 10.1111/jeb.13753

[evl3239-bib-0087] Lande, R. 1975. The maintenance of genetic variability by mutation in a polygenic character with linked loci. Genet Res 26:221–235. 1225762 10.1017/s0016672300016037

[evl3239-bib-0088] ———. 1980. Sexual dimorphism, sexual selection, and adaptation in polygenic characters. Evolution 34:292–305. 28563426 10.1111/j.1558-5646.1980.tb04817.x

[evl3239-bib-0089] ———. 1981. Models of speciation by sexual selection on polygenic traits. Proc Natl Acad Sci USA 78:3721–3725. 16593036 10.1073/pnas.78.6.3721 PMC319643

[evl3239-bib-0158] Lande, R. , and S. Arnold . 1983. The measurement of selection on correlated characters. Evolution 37:1210–1226. 28556011 10.1111/j.1558-5646.1983.tb00236.x

[evl3239-bib-0090] Lehtonen, J. , M. D. Jennions , and H. Kokko 2012. The many costs of sex. Trends Ecol Evol 27:172–178. 22019414 10.1016/j.tree.2011.09.016

[evl3239-bib-0091] Lenarcic, A. B. , K. L. Svenson , G. A. Churchill , and W. Valdar 2012. A general Bayesian approach to analyzing diallel crosses of inbred strains. Genetics 190:413–435. 22345610 10.1534/genetics.111.132563 PMC3276624

[evl3239-bib-0092] Long, T. A. , A. F. Agrawal , and L. Rowe 2012. The effect of sexual selection on offspring fitness depends on the nature of genetic variation. Curr Biol 22:204–208. 22226747 10.1016/j.cub.2011.12.020

[evl3239-bib-0093] Lorch, P. D. , S. Proulx , L. Rowe , and T. Day 2003. Condition‐dependent sexual selection can accelerate adaptation. Evol Ecol Res 5:867–881.

[evl3239-bib-0094] Lumley, A. J. , Ł. Michalczyk , J. J. Kitson , L. G. Spurgin , C. A. Morrison , J. L. Godwin , et al. 2015. Sexual selection protects against extinction. Nature 522:470–473. 25985178 10.1038/nature14419

[evl3239-bib-0095] Lynch, M. , and B. Walsh 1998. Genetics and analysis of quantitative traits. Sinauer, Sunderland, MA.

[evl3239-bib-0096] Lynch, M. , J. Blanchard , D. Houle , T. Kibota , S. Schultz , L. Vassilieva , et al. 1999. Perspective: spontaneous deleterious mutation. Evolution 53:645–663. 28565627 10.1111/j.1558-5646.1999.tb05361.x

[evl3239-bib-0097] Maklakov, A. A. , and G. Arnqvist 2009. Testing for direct and indirect effects of mate choice by manipulating female choosiness. Curr Biol 19:1903–1906. 19853448 10.1016/j.cub.2009.08.058

[evl3239-bib-0098] Mallet, M. A. , and A. K. Chippindale 2011. Inbreeding reveals stronger net selection on *Drosophila melanogaster* males: implications for mutation load and the fitness of sexual females. Heredity 106:994–1002. 21119701 10.1038/hdy.2010.148 PMC3186252

[evl3239-bib-0099] Mallet, M. A. , J. M. Bouchard , C. M. Kimber , and A. K. Chippindale 2011. Experimental mutation‐accumulation on the X chromosome of *Drosophila melanogaster* reveals stronger selection on males than females. BMC Evol Biol 11:156. 21645375 10.1186/1471-2148-11-156 PMC3134001

[evl3239-bib-0100] Mallet, M. A. , C. M. Kimber , and A. K. Chippindale 2012. Susceptibility of the male fitness phenotype to spontaneous mutation. Biol Lett 8:426–429. 22090202 10.1098/rsbl.2011.0977 PMC3367732

[evl3239-bib-0101] Manning, J. T. 1984. Males and the advantage of sex. J Theor Biol 108:215–220. 6748688 10.1016/s0022-5193(84)80067-3

[evl3239-bib-0167] Martin, G. , S. F. Elena , and T. Lenormand . 2007. Distributions of epistasis in microbes fit predictions from a fitness landscape model. Nat. Genet. 39:555–560. 17369829 10.1038/ng1998

[evl3239-bib-0102] Martinossi‐Allibert, I. , C. Rueffler , G. Arnqvist , and D. Berger 2019a. The efficacy of good genes sexual selection under environmental change. Proc Biol Sci 286:20182313. 30963930 10.1098/rspb.2018.2313 PMC6408614

[evl3239-bib-0103] Martinossi‐Allibert, I. , E. Thilliez , G. Arnqvist , and D. Berger 2019b. Sexual selection, environmental robustness, and evolutionary demography of maladapted populations: a test using experimental evolution in seed beetles. Evol App 12:1371–1384. 10.1111/eva.12758 PMC6691221 31417621

[evl3239-bib-0104] Martinossi‐Allibert, I. , J. Liljestrand Rönn , and E. Immonen 2020. Female‐specific resource limitation does not make the opportunity for selection more female biased. Evolution 74:2714–2724. 33043452 10.1111/evo.14106 PMC7821317

[evl3239-bib-0105] Matsumura, S. , R. Arlinghaus , and U. Dieckmann 2012. Standardizing selection strengths to study selection in the wild: a critical comparison and suggestions for the future. BioSci 62:1039–1054.

[evl3239-bib-0106] Maurizio, P. L. , M. T. Ferris , G. R. Keele , D. R. Miller , G. D. Shaw , A. C. Whitmore , *et al*. 2018. Bayesian diallel analysis reveals *Mx1*‐dependent and *Mx1*‐independent effects on response to influenza A virus in mice. G3 8:427–445. 29187420 10.1534/g3.117.300438 PMC5919740

[evl3239-bib-0107] Maynard Smith, J. 1971. What use is sex? J Theor Biol 30:319–335. 5548029 10.1016/0022-5193(71)90058-0

[evl3239-bib-0108] ———. 1978. The evolution of sex. Cambridge Univ. Press, Cambridge, U.K.

[evl3239-bib-0109] McGlothlin, J. W. , R. M. Cox , and E. D. Brodie III 2019. Sex‐specific selection and the evolution of between‐sex genetic covariance. J Hered 110:422–432. 31095325 10.1093/jhered/esz031

[evl3239-bib-0110] Mitchell‐Olds, T. , J. H. Willis , and D. B. Goldstein 2007. Which evolutionary processes influence natural genetic variation for phenotypic traits? Nat Rev Genet 8:845–856. 17943192 10.1038/nrg2207

[evl3239-bib-0111] Miyatake, T. , and F. Matsumura 2004. Intra‐specific variation in female remating in *Callosobruchus chinensis* and *C. maculatus* . J Insect Physiol 50:403–408. 15121453 10.1016/j.jinsphys.2004.02.007

[evl3239-bib-0114] Parker, G. A. , R. R. Baker , and V. G. F. Smith 1972. The origin and evolution of gamete dimorphism and the male‐female phenomenon. J Theor Biol 36:529–553. 5080448 10.1016/0022-5193(72)90007-0

[evl3239-bib-0115] Pischedda, A. , and A. K. Chippindale 2006. Intralocus sexual conflict diminishes the benefits of sexual selection. PLoS Biol 4:e356. 17105343 10.1371/journal.pbio.0040356 PMC1618422

[evl3239-bib-0116] Pitnick, S. , M. F. Wolfner , and S. S. Suarez 2009. Ejaculate‐female and sperm‐female interactions. In: (Sperm biology: an evolutionary perspective) {eds. Birkhead, T.R. , Hosken, D.J. & Pitnick, S.S. }. Elsevier, Oxford, U.K., pp.247–304.

[evl3239-bib-0117] Postma, E. 2006. Implications of the difference between true and predicted breeding values for the study of natural selection and micro‐evolution. J Evol Biol 19:309–320. 16599906 10.1111/j.1420-9101.2005.01007.x

[evl3239-bib-0118] ———. 2011. Comment on “Additive genetic breeding values correlate with the load of partially deleterious mutations. Science 333:1221–1221. 21885758 10.1126/science.1200996

[evl3239-bib-0121] Prokop, Z. M. , Ł. Michalczyk , S. M. Drobniak , M. Herdegen , and J. Radwan 2012. Meta‐analysis suggests choosy females get sexy sons more than “good genes. Evolution 66:2665–2673. 22946794 10.1111/j.1558-5646.2012.01654.x

[evl3239-bib-0122] R Core Team . 2019. R: a language and environment for statistical computing. R Foundation for Statistical Computing, Vienna. Available via https://www.R‐project.org/.

[evl3239-bib-0123] Radwan, J. 2004. Effectiveness of sexual selection in removing mutations induced with ionizing radiation. Ecol Lett 7:1149–1154.

[evl3239-bib-0159] Rausher, M. D 1992. The Measurement of Selection on Quantitative Traits: Biases Due to Environmental Covariances between Traits and Fitness. Evolution 46:616–626. 28568666 10.1111/j.1558-5646.1992.tb02070.x

[evl3239-bib-0125] Robertson, A. 1952. The effect of inbreeding on the variation due to recessive genes. Genetics 37:189–207. 17247385 10.1093/genetics/37.2.189 PMC1209550

[evl3239-bib-0128] Rose, M. R. 1982. Antagonistic pleiotropy, dominance, and genetic variation. Heredity 48:63–78.

[evl3239-bib-0129] Rowe, L. , and D. Houle 1996. The lek paradox and the capture of genetic variance by condition dependent traits. Proc Biol Sci 263:1415–1421.

[evl3239-bib-0130] Savalli, U. M. , and C. W. Fox 1999. The effect of male size, age, and mating behavior on sexual selection in the seed beetle *Callosobruchus maculatus* . Ethol Ecol Evol 11:49–60.

[evl3239-bib-0131] Sayadi, A. , A. M. Barrio , E. Immonen , J. Dainat , D. Berger , C. Tellgren‐Roth , et al. 2019. The genomic footprint of sexual conflict. Nat Ecol Evol 3:1725–1730. 31740847 10.1038/s41559-019-1041-9

[evl3239-bib-0132] Schärer, L. , L. Rowe , and G. Arnqvist 2012. Anisogamy, chance and the evolution of sex roles. Trends Ecol Evol 27:260–264. 22277154 10.1016/j.tree.2011.12.006

[evl3239-bib-0133] Sharp, N. P. , and A. F. Agrawal 2008. Mating density and the strength of sexual selection against deleterious alleles in *Drosophila melanogaster* . Evolution 62:857–867. 18221380 10.1111/j.1558-5646.2008.00333.x

[evl3239-bib-0134] ———. 2013. Male‐biased fitness effects of spontaneous mutations in *Drosophila melanogaster* . Evolution 67:1189–1195. 23550766 10.1111/j.1558-5646.2012.01834.x

[evl3239-bib-0135] ———. 2018. An experimental test of the mutation‐selection balance model for the maintenance of genetic variance in fitness components. Proc Biol Sci 285:20181864. 30404880 10.1098/rspb.2018.1864 PMC6235037

[evl3239-bib-0136] Shorter, J. R. , P. L. Maurizio , T. A. Bell , G. D. Shaw , D. R. Miller , T. J. Gooch , et al. 2019. A diallel of the mouse Collaborative Cross founders reveals strong strain‐specific maternal effects on litter size. G3 9:1613–1622. 30877080 10.1534/g3.118.200847 PMC6505174

[evl3239-bib-0137] Siller, S. 2001. Sexual selection and the maintenance of sex. Nature 411:689. 11395770 10.1038/35079578

[evl3239-bib-0138] Singh, A. , and D. Punzalan 2018. The strength of sex‐specific selection in the wild. Evolution 72:2818–2824. 30298925 10.1111/evo.13625

[evl3239-bib-0139] Singh, A. , A. F. Agrawal , and H. D. Rundle 2017. Environmental complexity and the purging of deleterious alleles. Evolution 71:2714–2720. 28840604 10.1111/evo.13334

[evl3239-bib-0140] Southgate, B. J. 1979. Biology of the Bruchidae. Annu Rev Entomol 24:449–473.

[evl3239-bib-0141] Spencer, H. G. , and N. K. Priest 2016. The evolution of sex‐specific dominance in response to sexually antagonistic selection. Am Nat 187:658–666. 27104997 10.1086/685827

[evl3239-bib-0142] Stewart, A. D. , A. Pischedda , and W. R. Rice 2010. Resolving intralocus sexual conflict: genetic mechanisms and time frame. J Hered 101:S94–S99. 20421329 10.1093/jhered/esq011 PMC2859891

[evl3239-bib-0143] Sztepanacz, J. L. , and D. Houle 2019. Cross‐sex genetic covariances limit the evolvability of wing‐shape within and among species of *Drosophila* . Evolution 73:1617–1633. 31206655 10.1111/evo.13788

[evl3239-bib-0144] Thornhill, R. 1983. Cryptic female choice and its implications in the scorpionfly *Harpobittacus nigriceps* . Am Nat 122:765–788.

[evl3239-bib-0145] Tomkins, J. L. , J. Radwan , J. S. Kotiaho , and T. Tregenza 2004. Genic capture and resolving the lek paradox. Trends Ecol Evol 19:323–328. 16701278 10.1016/j.tree.2004.03.029

[evl3239-bib-0146] Van Doorn, G. S. 2009. Intralocus sexual conflict. Ann NY Acad Sci 1168:52–71. 19566703 10.1111/j.1749-6632.2009.04573.x

[evl3239-bib-0147] Wade, M. J. 1979. Sexual selection and variance in reproductive success. Am Nat 114:742–747. 10.1086/424531 15459886

[evl3239-bib-0148] Wade, M. J. , and S. J. Arnold 1980. The intensity of sexual selection in relation to male sexual behaviour, female choice, and sperm precedence. Anim Behav 28:446–461.

[evl3239-bib-0149] Walsh, B. , and M. Lynch 2018. Theorems of natural selection: results of Price, Fisher, and Robertson. In: (Evolution and selection of quantitative traits), {eds. Walsh, B. , Lynch, M. }. Cambridge Univ. Press, Cambridge, U.K., pp. 145–172.

[evl3239-bib-0150] Whitlock, M. C. 2002. Selection, load and inbreeding depression in a large metapopulation. Genetics 160:1191–1202. 11901133 10.1093/genetics/160.3.1191 PMC1462034

[evl3239-bib-0151] Whitlock, M. C. , and A. F. Agrawal 2009. Purging the genome with sexual selection: reducing mutation load through selection on males. Evolution 63:569–582. 19154364 10.1111/j.1558-5646.2008.00558.x

[evl3239-bib-0169] Whitlock, M. C. , P. C. Phillips , F. B. G. Moore , and S. J. Tonsor . 1995. Multiple fitness peaks and epistasis. Annu. Rev. Ecol. Syst. 26:601–629.

[evl3239-bib-0152] Whitlock, M. C. , and B. Davis 2011. Genetic Load. eLS 10.1002/9780470015902.a0001787.pub2.

[evl3239-bib-0170] Wyman, M. J. , A. F. Agrawal , and L. Rowe . 2010. Condition‐dependence of the sexually dimorphic transcriptome in *Drosophila melanogaster* . Evolution 64:1836–1848. 20059540 10.1111/j.1558-5646.2009.00938.x

[evl3239-bib-0153] Yun, L. , P. J. Chen , A. Singh , A. F. Agrawal , and H. D. Rundle 2017. The physical environment mediates male harm and its effect on selection in females. Proc Biol Sci 284:20170424. 28679725 10.1098/rspb.2017.0424 PMC5524491

[evl3239-bib-0154] Yun, L. , P. J. Chen , K. E. Kwok , C. S. Angell , H. D. Rundle , and A. F. Agrawal 2018. Competition for mates and the improvement of nonsexual fitness. Proc Natl Acad Sci USA 115:6762–6767. 29891650 10.1073/pnas.1805435115 PMC6042133

[evl3239-bib-0155] Zahavi, A. 1975. Mate selection—a selection for a handicap. J Theor Biol 53:205–214. 1195756 10.1016/0022-5193(75)90111-3

[evl3239-bib-0156] Zhang, X. S. , J. Wang , and W. G. Hill 2004. Influence of dominance, leptokurtosis and pleiotropy of deleterious mutations on quantitative genetic variation at mutation‐selection balance. Genetics 166:597–610. 15020447 10.1534/genetics.166.1.597 PMC1470700

