# Supplementary material for: Selection in males purges the mutation load on female fitness
Source: Evol Lett. 2021 Jun 26;5(4):328–43. doi: 10.1002/evl3.239 (PMC8327962; doi:10.1002/evl3.239)
Supplement: Supplementary file 1 — Table S1. Comparison and rationale for using opposite‐sex heterosis for point estimates and sex‐averaged heterosis for sex‐differences. Fig. S1. Lome population history and experimental design. Fig. S2. The raw‐means r MF of the isofemale lines. Fig. S3. Results the Gelman‐Rubin analysis, demonstrating good mixing of four independent MCMC chains. Fig. S4. Outbred (o) breeding values from Fig. 1A shaded by sex‐averaged heterosis. Fig. S5. Inbred (i) breeding values from Fig. 1A shaded by sex‐averaged heterosis. Fig. S6. Resampled point estimates and 95% credibility intervals of βa′ for males (βaM′) and females (βaF′). [file EVL3-5-328-s001.pdf]

Supporting Information for:

Selection in males purges the mutation load on female fitness

Karl Grieshop[1,2,3], Paul L. Maurizio[4], Göran Arnqvist[1] and David Berger[1]

[1]Department of Ecology and Genetics, Animal Ecology, Uppsala University, Uppsala, Sweden

[2]Department of Ecology and Evolutionary Biology, University of Toronto, Toronto, Canada

[3]Department of Molecular Biosciences, The Wenner-Gren Institute, Stockholm University, Stockholm, Sweden

[4]Section of Genetic Medicine, Department of Medicine, University of Chicago, Chicago, Illinois, United States

Correspondence:
Karl Grieshop
Ecology and Evolutionary Biology
University of Toronto
25 Willcocks St, Toronto, ON M5S 3B2, Canada
Phone: +1 647 230 5077
karlgrieshop@gmail.com

Table S1: Comparison and rationale for using opposite-sex heterosis for point estimates and sex-averaged heterosis for sex-differences. Using opposite-sex heterosis is the most technically correct approach for the point estimates and assessment of whether they are different from zero, but causes male and female estimates to not be directly comparable. Using sex-averaged heterosis renders the sex-specific estimates directly comparable – and is therefore the most technically correct assessment of fold differences and significance between the sexes – but the estimates may be biased due to shared measurement error between fitness and heterosis. Estimates and values used to support the conclusions are bolded.

Description	Heterosis	Sex	Symbol	Estimate (CIs)	*P* value	Fold difference	Sex-diff. *P* value
Genetic standardized selection	Opposite-sex	**Male**	$\boldsymbol{\beta'_{a_M}}$	**-0.0125 (-0.031, -0.003)**	**0.008**	8.7x	0.11
		**Female**	$\boldsymbol{\beta'_{a_F}}$	**-0.0014 (-0.013, 0.009)**	**0.672**		
	Sex-averaged	Male	β''_{a_M}	-0.0133 (-0.029, -0.004)	0.008	**3.7x**	**0.104**
		Female	β''_{a_F}	-0.0036 (-0.011, 0.007)	0.558		
Genetic correlation	Opposite-sex	**Male**	$\boldsymbol{r_{o_M,\, o_F-i_F}}$	**-0.59 (-0.81, -0.11)**	**0.008**	-	0.132
		**Female**	$\boldsymbol{r_{o_F,\, o_M-i_M}}$	**-0.14 (-0.40, 0.28)**	**0.672**		
	Sex-averaged	Male	-	-0.46 (-0.79, -0.12)	0.008	-	**0.12**
		Female	-	-0.06 (-0.37, 0.17)	0.558		

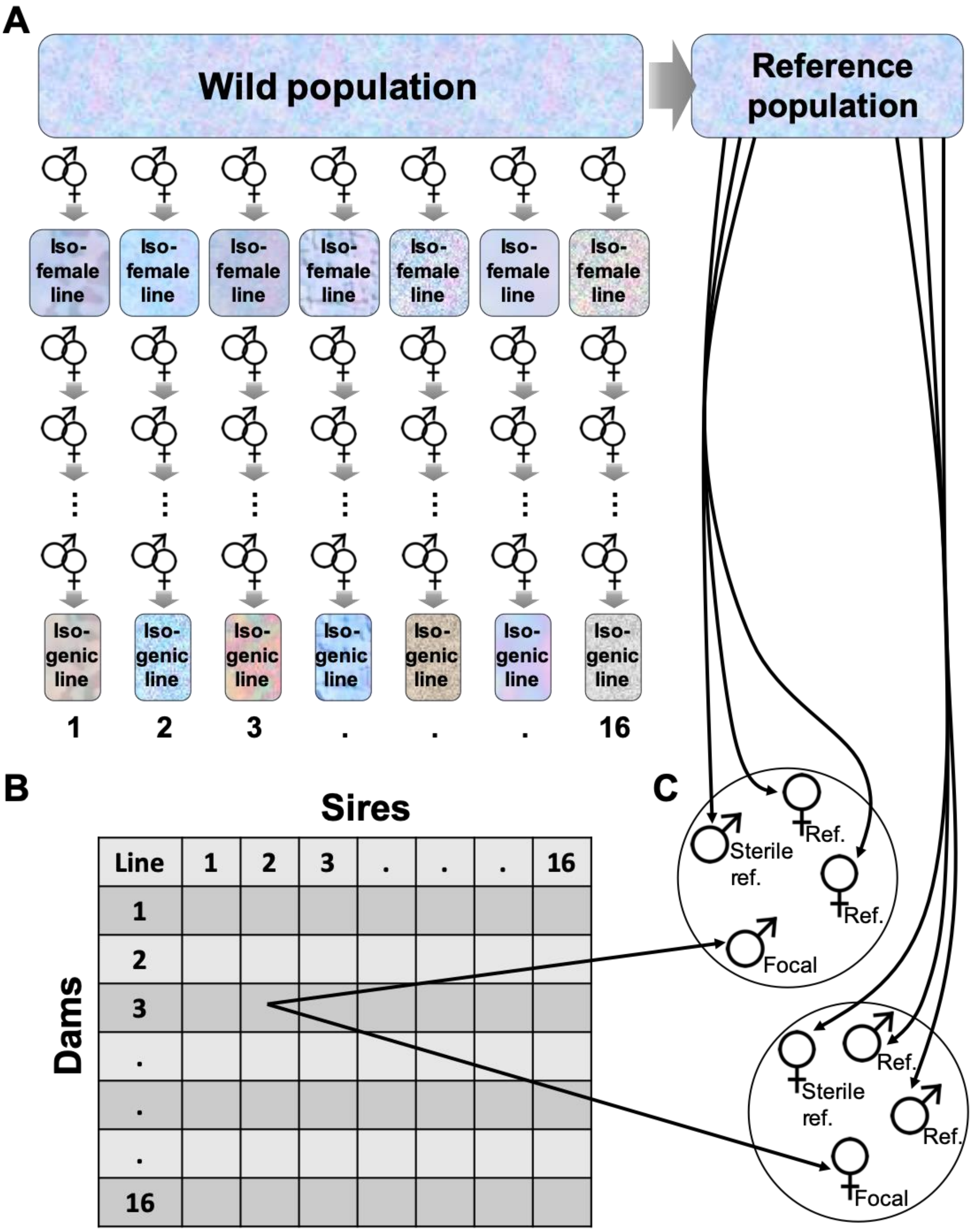

Fig. S1: Lome population history and experimental design. (A) A wild population was divided into an outbred laboratory reference population and 41 isofemale lines (Berger et al. 2014). The latter were further inbred (Grieshop et al. 2017) to obtain the 16 inbred/isogenic lines used presently (Grieshop and Arnqvist 2018). Genetic diversity is depicted by color and texture. (B) A full diallel cross (Lynch and Walsh 1998) among the 16 inbred strains, where F_1 inbred parental selfs (*i*) are on the diagonal and outcrossed F_1s (*o*) are on the off-diagonal. (C) Replicate F_1 male and female fitness estimates included a focal F_1 individual (e.g. outbred (*o*) F_1s from a strain-2 sire and strain-3 dam), a sterile same-sex competitor from the reference population, two (fertile) opposite-sex competitors from the reference population, and ca. 100 *V. unguiculate* seeds in a 90 mm ø petri dish. These beetles were left to interact/mate/oviposit for the duration of their lifetime, and F_2 offspring counts = focal individuals' fitness.

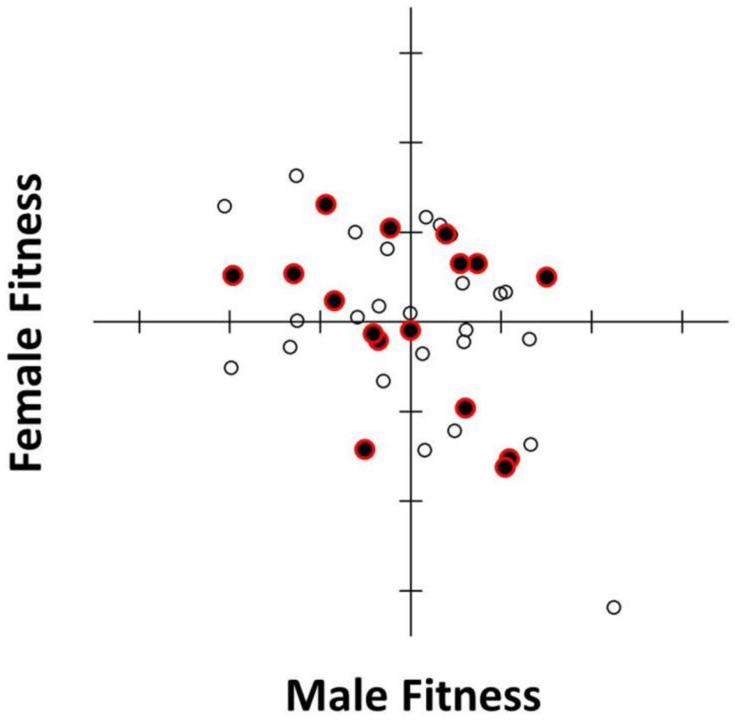

Fig. S2: The raw-means r_{MF} of the isofemale lines (see Fig. S1; Berger et al. 2014). Those from whence the present 16 inbred strains stem are outlined in red and filled in black. Because all 20 replicate inbreeding lineages stemming from some of the most male-benefit/female-detriment isofemale lines went extinct prior to completing the full inbreeding program (Grieshop et al. 2017), the present 16 inbred strains were chosen from throughout the isofemale line r_{MF} with the aim of countering that bias.

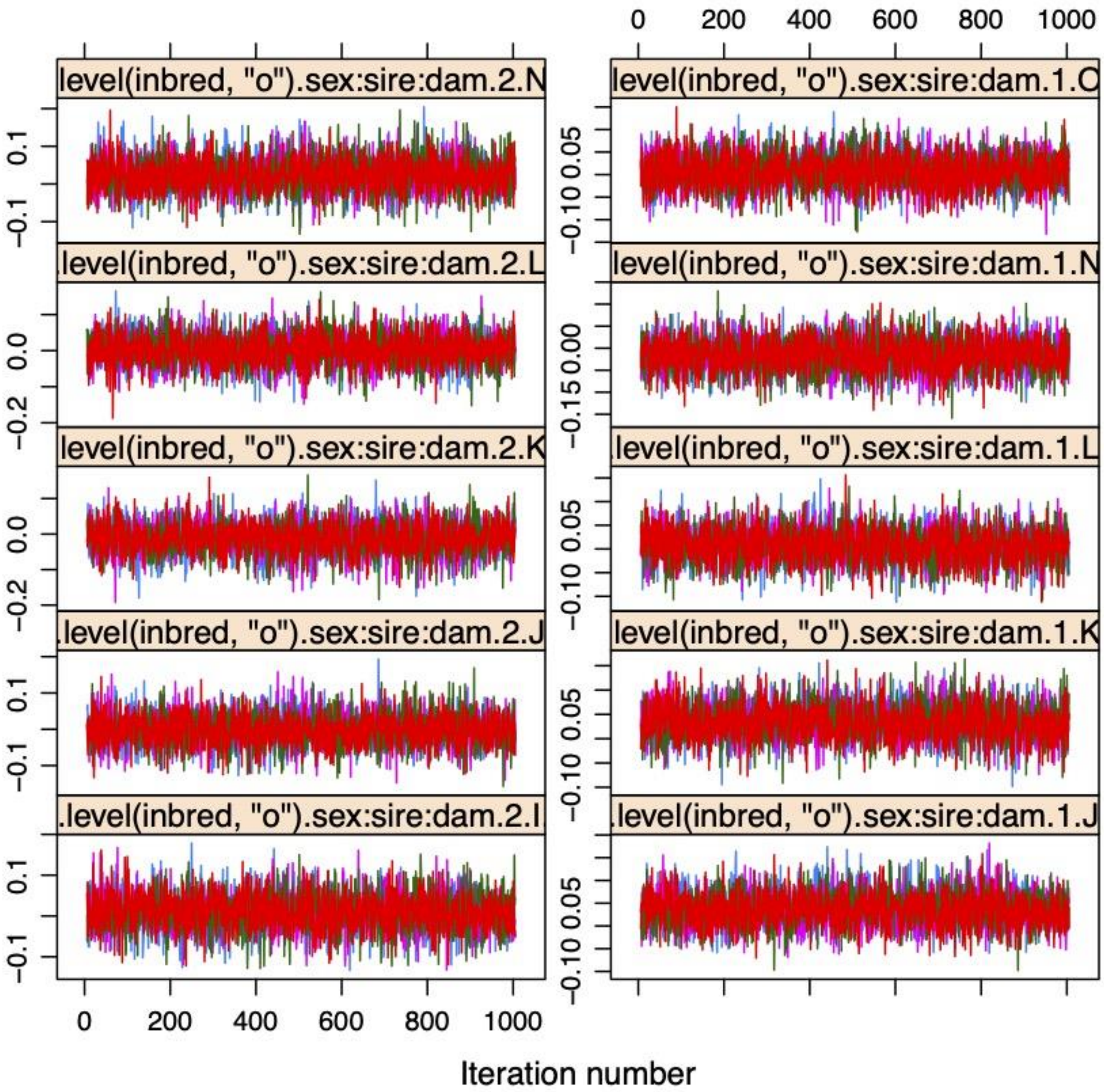

Fig. S3: Results the Gelman-Rubin analysis, demonstrating good mixing of four independent MCMC chains. Shown is a representative random sample of ten parameters, out of the total 780 parameters (plus an intercept) that were estimated in our model. Other diagnostic output from this analysis is available in the R script (starting on line 110).

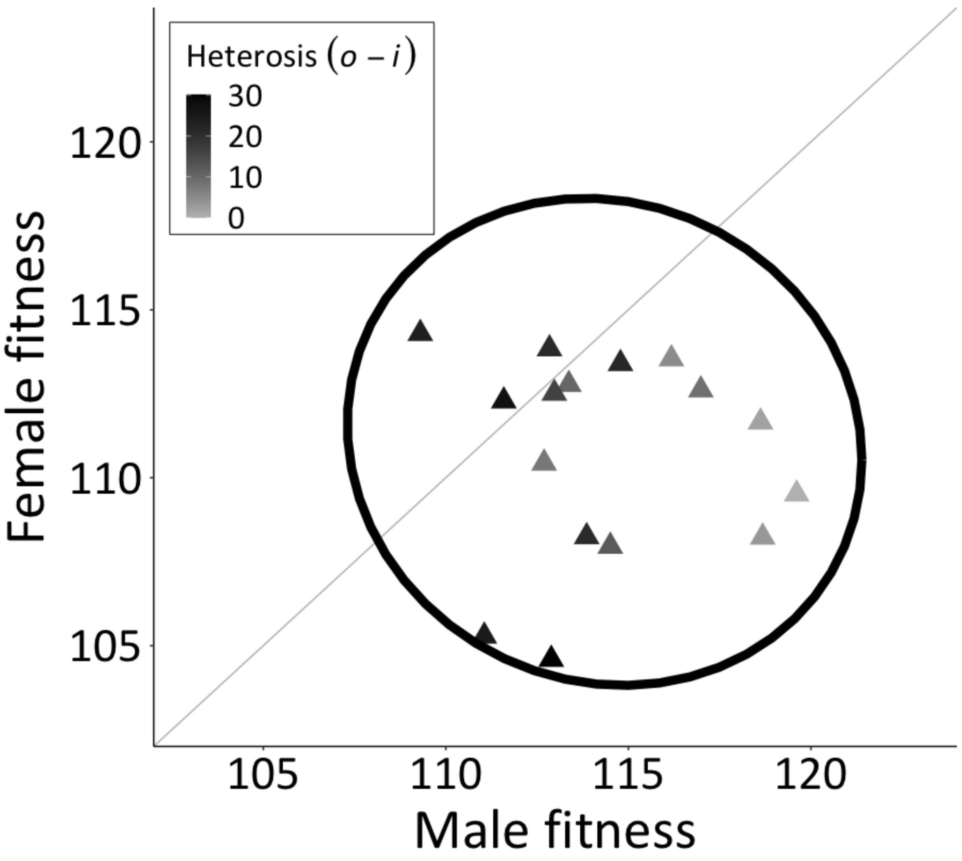

Fig. S4: Outbred (*o*) breeding values from Fig. 1A shaded by sex-averaged heterosis. An informal depiction of our main finding is apparent in that heterosis is clearly distributed along a horizontal gradient, according to male breeding values for fitness, but is clearly not distributed along the vertical dimension. Thus, sex-/strain-specific heterosis, the degree to which inbred strains benefit from having their rare partially recessive deleterious mutations covered up by heterozygosity, is reflected in those strains' outcrossed males, but not their outcrossed females.

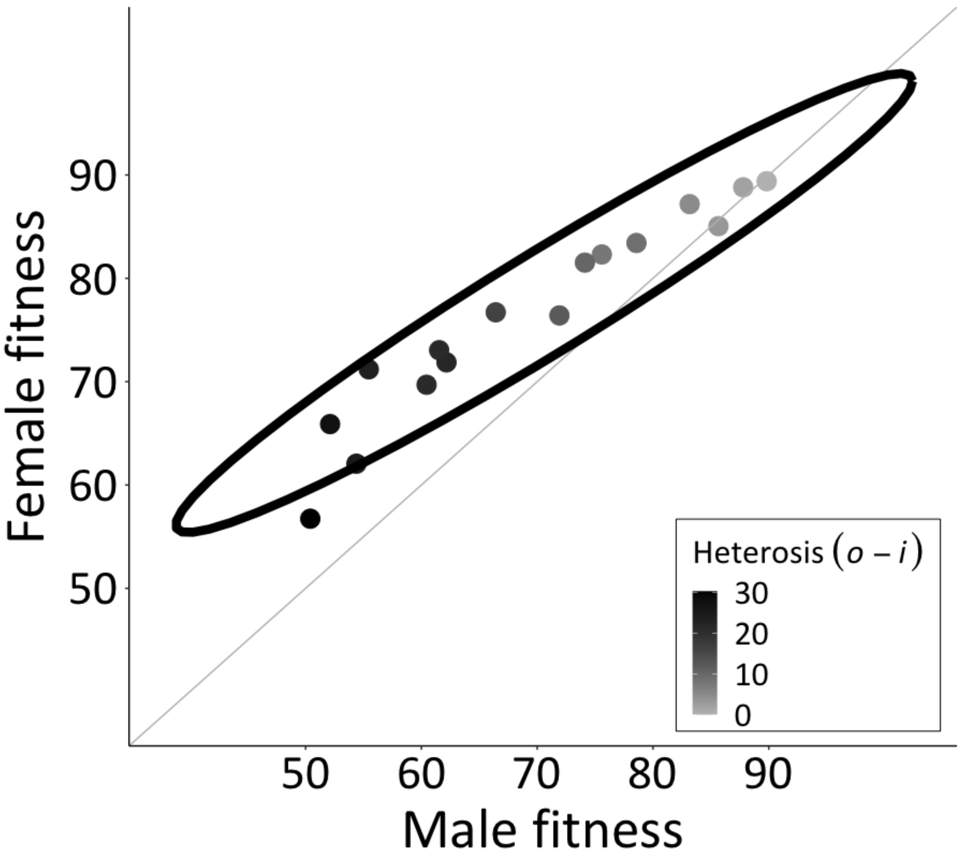

Fig. S5: Inbred (*i*) breeding values from Fig. 1A shaded by sex-averaged heterosis. By definition, strains with greater inbred fitness experience less heterosis. Male fitness is more negatively impacted by inbreeding/homozygosity than female fitness, as the large majority of these strains' inbred breeding values lie above the y=x line.

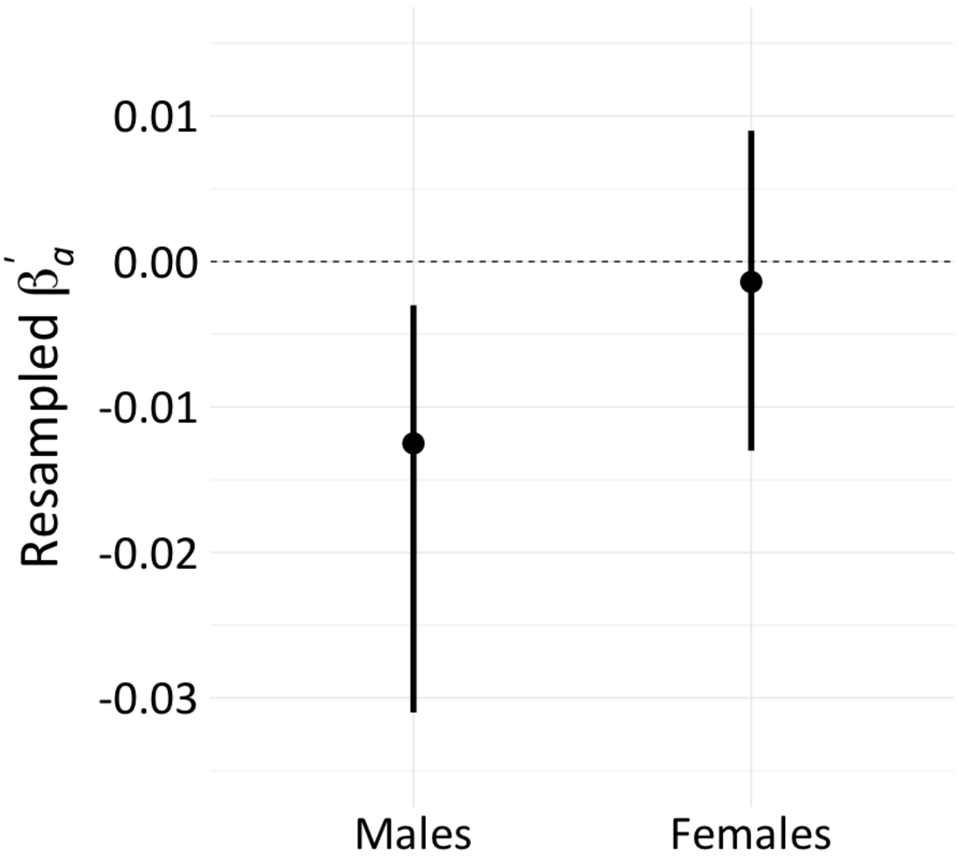

Fig. S6: Resampled point estimates and 95% credibility intervals of β'_a for males (β'_{a_M}) and females (β'_{a_F}).

SI References

Berger, D., Grieshop, K., Lind, M.I., Goenaga, J., Maklakov, A.A. and Arnqvist, G. (2014). Intralocus sexual conflict and environmental stress. *Evolution* 68:2184-2196.

Grieshop, K., Berger, D. and Arnqvist, G. (2017). Male-benefit sexually antagonistic genotypes show elevated vulnerability to inbreeding. *BMC Evol Biol* 17:134.

Grieshop, K. and Arnqvist, G. (2018). Sex-specific dominance reversal of genetic variation for fitness. *PLoS Biol* 16:e2006810.
